# A Machine Learning Model for AA7075-T6 Based on Anisotropic Elastoplastic Constitutive Theory

**DOI:** 10.3390/ma19194165

**Published:** 2026-09-29

**Authors:** Lin Lv, Fuxing Ye, Tao Jin, Hui Lin

**Affiliations:** 1 School of Materials Science and Engineering, Tianjin University, Tianjin 310018, China; lvlinzipc@163.com; 2 School of Geely Automobile, Hangzhou Polytechnic University, Hangzhou 310018, China; 3 School of Intelligent Manufacturing, Taizhou University, Taizhou 318000, China; tyutjintao@163.com

**Keywords:** AA7075-T6, anisotropic constitutive model, full stress state, stress-strain relation, machine learning

## Abstract

In this study, a machine learning model for the anisotropic mechanical behavior of AA7075-T6 was successfully developed by combining classical plasticity theory with neural network algorithms. First, a yield criterion incorporating strength asymmetry and orientation dependence was constructed based on a fourth-order linear transformation of invariants, and a stress update algorithm was implemented using the return mapping and implicit integration schemes. Subsequently, a dataset comprising experimental results from tension, compression, and shear tests at multiple orientations, as well as theoretically generated data from additional strain paths, was established to train a genetic algorithm-optimized two-hidden-layer neural network. Plastic-stage assessments indicated that, for the equal-biaxial path, the RMSE values of the stress–strain curves predicted by the machine learning model relative to the constitutive implementation were 31.87 and 39.24 MPa. It should be noted that the stress–strain response under equal-biaxial loading represents an additional prediction case. The trained model exhibited satisfactory predictive performance when compared against experimental data for tension, compression, and shear and was capable of reproducing the theoretical stress paths derived from the classical constitutive model. This approach leverages the physical interpretability of conventional constitutive modeling and the high efficiency of data-driven methods, providing a viable solution for efficiently predicting the anisotropic mechanical response of metallic materials under complex stress states.

## 1. Introduction

Aluminum alloys are widely used in automotive, construction, aerospace, and aviation industries due to their high specific stiffness, high specific strength, and favorable corrosion resistance [1,2,3]. To date, the mechanical properties of AA7075-T6 have been extensively investigated [4,5,6,7,8,9]. Some researchers have focused on the mechanical behavior of the material under quasi-static loading [10,11,12], dynamic loading [13,14,15], explosive loading [16], various temperatures [17,18], fatigue loading [19], and complex stress states [18,20,21,22,23,24]. A comprehensive understanding of the material’s mechanical response under different loading conditions facilitates the development of mechanical constitutive models and supports material selection for load-bearing or structural components [25].

In recent years, with the advent of the big data era and the continuous development and refinement of various optimization algorithms, data-driven and machine learning (ML)-based strategies have been proposed and developed for modeling the mechanical behavior of materials and structures [26,27,28,29,30]. Liu et al. compared fracture toughness predictions obtained from analytical, empirical, and ML methods using regression trees and neural networks and found that the predictions from regression trees and neural networks were in good agreement with experimental results, demonstrating the reliability and rapid deployment capability of ML methods in material fracture toughness prediction [31]. Similarly, Reuter et al. employed ML algorithms such as artificial neural networks (ANN), support vector machines (SVM), and support vector regression (SVR) to construct failure surfaces for concrete, aiming to develop model-free approaches for building strength models for new types of concrete and thus avoid repeated modeling using traditional strength criteria [32]. Based on similar considerations and incorporating strain-dependent plastic behavior, Mozaffar et al. [33] utilized sequence learning of recurrent neural networks to identify constitutive models dependent on loading history and microstructure, thereby circumventing the three fundamental assumptions of traditional theory: yield criteria, flow rules, and the principle of plastic work equivalence [33]. Liu et al. investigated the in-plane nonlinear shear constitutive behavior and failure initiation criteria of fiber-reinforced composites using neural networks [34]. Gorji et al. developed a neural network model to replace the traditional complex return mapping framework and applied it to describe the large deformation behavior of von-Mises materials under isotropic hardening assumptions [35]. In short, this model-free approach to constructing material strength criteria offers a flexible research method that overcomes the limitations of traditional methods in terms of generalizability.

To avoid the loss of physical meaning in ML models, Wen et al. innovatively incorporated ML methods to obtain the plastic multiplier based on the consistency condition and hardening modulus, while still adhering to the traditional constitutive modeling framework. This led to a physically driven ML-based constitutive model for lithium metal over a wide temperature range and a large strain rate range [36]. This approach integrates traditional modeling concepts with ML methods, optimizing computational efficiency while preserving the physical significance of the strength model. Similarly, Jordan et al. [37] proposed a hybrid modeling approach combining mechanistic and data-driven modeling. In their work, the “big data” required for the ML model were generated using a custom-made robot-assisted testing system. Without any a priori assumptions on the specific mathematical form, ML methods were used to determine the functional relationship between stress and viscous strain, viscous strain rate, and temperature. A Bayesian regularized backpropagation algorithm was employed to determine the appropriate neural network function. The results demonstrated that the developed hybrid model reliably predicted the material’s stress–strain response. Other researchers have combined ANN with crystal plasticity finite element methods at the mesoscale to predict the stress–strain response and texture evolution of AA6063-T9 under uniaxial tension and shear loading conditions through model training [38]. Driven by the need for efficient solutions, combining data-driven approaches with ML is one of the preferred strategies for modeling the mechanical response of materials [32,33,36,39,40,41,42,43,44].

Furthermore, physically motivated ML models have also been applied to address mechanical problems arising from engineering system studies. Samaniego et al. [39] explored deep neural networks (DNNs) as function approximators to solve partial differential equations (PDEs). They analyzed different energy forms of PDEs, particularly the variational energy form, and reformulated them as loss functions for DNNs, demonstrating the effectiveness of combining energy methods with DNNs to solve PDEs in engineering applications. Based on this concept, Nguyen-Thanh et al. [45] proposed a deep energy method to account for strain gradient effects in elasticity. Similar to the isoparametric finite element method, a parameter space was defined, and model training was performed within this space. Compared with the original deep energy method, naturally normalized inputs not only prevented gradient vanishing but also led to faster convergence. Additionally, Goswami et al. [39] developed a physics-informed neural network algorithm that minimizes the variational energy of mechanical systems and applied it to address brittle fracture problems. These studies have inspired solution strategies for yield function-related problems.

Based on the above review, most existing studies focus on the stress–strain response of thin metal sheets under simple loading conditions and often involve simplifying assumptions. However, plastic anisotropy is a critical factor that cannot be overlooked when analyzing the mechanical behavior of sheet metals. Considering these factors, the development of stress response prediction models based on artificial neural networks holds significant potential for practical applications.

## 2. Anisotropic Plastic Constitutive Relation

To establish a full-stress-state constitutive model that captures the anisotropic mechanical behavior of metal sheets, it is necessary to comprehensively account for both the elastic and plastic behaviors of the material. Based on the experimental results of the stress–strain response reported in the previous section and considering the simplicity of algorithm implementation, the elastic behavior is described using isotropic elasticity. Since the mechanical properties of metal sheets primarily focus on the in-plane anisotropy within the RD-TD plane [44], a two-dimensional constitutive framework is adopted. For the anisotropic plastic behavior, a fourth-order linear transformation of invariants is introduced, and an odd function formulation is further incorporated to account for strength asymmetry (i.e., shear anisotropy).

### 2.1. Isotropic Elastic Rule

For the AA7075-T6 sheet, the two-dimensional (2D) isotropic elastic rule can be written as:

(1) $$\sigma_{ij}=C_{ijkl}\varepsilon_{kl}$$

(2) $$\sigma_{ij}={\left[\begin{array}{ccc} \sigma_{xx} & \sigma_{yy} & \sigma_{xy} \end{array}\right]}^{T}$$

(3) $$\varepsilon_{ij}={\left[\begin{array}{ccc} \varepsilon_{xx} & \varepsilon_{yy} & \varepsilon_{xy} \end{array}\right]}^{T}$$

For the 2D case, the elastic modulus tensor can be expressed as:

(4) $$\left[C\right]=\left[\begin{array}{ccc} \frac{E}{\left(1-\upsilon^{2}\right)} & \frac{E\upsilon }{\left(1-\upsilon^{2}\right)} & 0\\ \frac{E\upsilon }{\left(1-\upsilon^{2}\right)} & \frac{E}{\left(1-\upsilon^{2}\right)} & 0\\ 0 & 0 & \frac{E}{1+\upsilon } \end{array}\right]$$

The values of elastic modulus and Poisson’s ratio are set to be *E* = 71.7 GPa and υ = 0.33.

### 2.2. Return Mapping

To achieve stress updating under full stress state conditions, a rate-form integration algorithm of the constitutive equations, such as the return mapping algorithm, is commonly used:

(5) $$\begin{array}{l} \dot{\sigma }=C:{\dot{\varepsilon }}^{e}(\dot{\varepsilon }-{\dot{\varepsilon }}^{p})\\ {\dot{\varepsilon }}^{p}=\dot{\lambda }g\\ q=\dot{\lambda }h\\ \dot{f}=f_{\sigma }:\dot{\sigma }+f_{q}\cdot \dot{q}=0 \end{array}$$

where $\dot{\lambda }$, g, q, h, $f_{\sigma }$, $f_{q}$ are respectively denote the plastic multiplier, flow direction, internal variable, hardening modulus, the partial derivative of the yield function with respect to the stress tensor, and the partial derivative with respect to the internal variable. The so-called return mapping algorithm primarily refers to the process in which, after the material enters the plastic stage, the stress update at each time step is first integrated based on the elastic predictor. Subsequently, the yield criterion is used to determine whether the material is in the elastic or plastic state. If elastic, the integrated value is directly adopted; otherwise, based on the consistency condition and flow rule, the value obtained from the elastic predictor is mapped back onto the yield surface. This is therefore termed the return mapping algorithm.

### 2.3. Implicit Integration Algorithm

For stress updates at the integration point level, commonly used algorithms primarily include the forward Euler explicit algorithm, the quasi-implicit algorithm, and the fully implicit algorithm. Among these, the forward Euler algorithm performs updates based on the converged values from the previous step. The quasi-implicit algorithm employs an explicit treatment for the plastic flow direction and the plastic modulus: the increment of the plastic parameter is calculated at the end of the step, whereas the plastic flow direction and the plastic modulus are evaluated at the beginning of the step. The fully implicit algorithm (also known as the backward Euler integration algorithm) calculates the flow direction and the plastic multiplier at the end of the current time step and enforces the yield condition at the end of the step. This approach avoids drift of the integrated stress state from the yield surface and has the advantage of being unconditionally stable. The specific algorithm can be described as follows [46]:

(6) $$\begin{array}{l} \varepsilon_{n+1}=\varepsilon_{n}+\Delta \varepsilon \\ {\varepsilon^{p}}_{n+1}=\varepsilon_{n}^{p}+\Delta \lambda_{n+1}g_{n+1}\\ q_{n+1}=q_{n}+\Delta \lambda_{n+1}h_{n+1}\\ \sigma_{n+1}=C:(\varepsilon_{n+1}-\varepsilon_{n+1}^{p})\\ f_{n+1}=f(\sigma_{n+1},q_{n+1})=0 \end{array}$$

To perform numerical integration, it is first necessary to discretize the above integral formulation. For simplicity of description, omitting the subscript n + 1 for the current time step and rearranging yields:

(7) $$\begin{array}{l} a=-\varepsilon^{p}+\varepsilon_{n}^{p}+\Delta \lambda g=0\\ b=-q+q_{n}+\Delta \lambda h=0\\ f=f_{Y}(\sigma ,q)=0 \end{array}$$

By linearizing the above system of equations using Taylor expansion:

(8) $$\begin{array}{l} a^{\left(k\right)}+C^{-1}:\Delta \sigma^{\left(k\right)}+\Delta \lambda^{\left(k\right)}(g_{\sigma }^{\left(k\right)}:\Delta \sigma^{\left(k\right)}+g_{q}^{\left(k\right)}\cdot \Delta q^{\left(k\right)})+\delta \lambda^{\left(k\right)}g^{\left(k\right)}=0\\ b^{\left(k\right)}-\Delta q^{\left(k\right)}+\Delta \lambda^{\left(k\right)}(h_{\sigma }^{\left(k\right)}:\Delta \sigma^{\left(k\right)}+h_{q}^{\left(k\right)}\cdot \Delta q^{\left(k\right)})+\delta \lambda^{\left(k\right)}h^{\left(k\right)}=0\\ f^{\left(k\right)}+f_{\sigma }^{\left(k\right)}:\Delta \sigma^{\left(k\right)}+f_{q}^{\left(k\right)}\cdot \Delta q^{\left(k\right)}=0 \end{array}$$

The superscript k denotes the iteration step, while the subscript denotes the partial derivative with respect to it. Further rearrangement yields:

(9) $$\begin{array}{l} (C^{-1}+\Delta \lambda^{\left(k\right)}g_{\sigma }^{\left(k\right)}):\Delta \sigma^{\left(k\right)}+\Delta \lambda^{\left(k\right)}g_{q}^{\left(k\right)}\cdot \Delta q^{\left(k\right)}=-a^{\left(k\right)}-\delta \lambda^{\left(k\right)}g^{\left(k\right)}\\ \Delta \lambda^{\left(k\right)}h_{\sigma }^{\left(k\right)}:\Delta \sigma^{\left(k\right)}-\Delta q^{\left(k\right)}+\Delta \lambda^{\left(k\right)}h_{q}^{\left(k\right)}\cdot \Delta q^{\left(k\right)}=-b^{\left(k\right)}-\delta \lambda^{\left(k\right)}h^{\left(k\right)}\\ f_{\sigma }^{\left(k\right)}:\Delta \sigma^{\left(k\right)}+f_{q}^{\left(k\right)}\cdot \Delta q^{\left(k\right)}=-f^{\left(k\right)} \end{array}$$

Thus, the following system of equations is obtained:

(10) $$\left[\begin{array}{ccc} C^{-1}+\Delta \lambda^{\left(k\right)}g_{\sigma }^{\left(k\right)} & \Delta \lambda^{\left(k\right)}g_{q}^{\left(k\right)} & g^{\left(k\right)}\\ \Delta \lambda^{\left(k\right)}h_{\sigma }^{\left(k\right)} & \Delta \lambda^{\left(k\right)}h_{q}^{\left(k\right)}-I & h^{\left(k\right)}\\ f_{\sigma }^{\left(k\right)} & f_{q}^{\left(k\right)} & o \end{array}\right]\left[\begin{array}{c} \Delta \sigma^{\left(k\right)}\\ \Delta q^{\left(k\right)}\\ \delta \lambda^{\left(k\right)} \end{array}\right]=\left[\begin{array}{c} -a^{\left(k\right)}\\ -b^{\left(k\right)}\\ -f^{\left(k\right)} \end{array}\right]$$

where o and I are the zero matrix and the identity matrix, respectively. For the combined anisotropic and isotropic hardening law to be adopted in this study, the internal variables q involve only the hardening parameter and the equivalent plastic strain and can be described as follows:

(11) $$q={\left[\begin{array}{cc} c_{i}(\varepsilon_{eq}^{p}) & \varepsilon_{eq}^{p} \end{array}\right]}^{T}$$

where the subscript i denotes the number of model parameters. Adopting the associated flow rule, it follows that:

(12) $$d\lambda =d\varepsilon_{eq}^{p}$$

The above system of equations can be rewritten as:

(13) $$\left[\begin{array}{ccc} C^{-1}+\Delta \lambda^{\left(k\right)}g_{\sigma }^{\left(k\right)} & \Delta \lambda^{\left(k\right)}g_{q}^{\left(k\right)} & g^{\left(k\right)}\\ o & -I & h^{\left(k\right)}\\ f_{\sigma }^{\left(k\right)} & f_{q}^{\left(k\right)} & f_{\varepsilon_{eq}^{p}}^{\left(k\right)} \end{array}\right]\left[\begin{array}{c} \Delta \sigma^{\left(k\right)}\\ \Delta q^{\left(k\right)} \end{array}\right]=\left[\begin{array}{c} -a^{\left(k\right)}\\ -b^{\left(k\right)}\\ -f^{\left(k\right)} \end{array}\right]$$

By solving the above system of equations, the increments of stress, internal variables, and state variables at each time step can be obtained, thereby achieving stress update at the integration point.

### 2.4. Anisotropic Yield Criterion

Considering the working conditions addressed in this study, a stress invariants-based criterion (SIM) is employed to develop a constitutive model that captures both plastic anisotropy and strength asymmetry to describe the mechanical behavior of the AA7075-T6 sheet metal [46,47]:

(14) $$f(\sigma )={\left(J_{2}^{\prime }(s^{\prime })\right)}^{3/2}-J_{3}^{\prime \prime }(s^{\prime \prime })-\tau_{s}^{3}$$

(15) $$\left\{\begin{array}{l} s^{\prime }=L^{\prime }\sigma \\ s^{\prime \prime }=L^{\prime \prime }\sigma \end{array}\right.$$

where the invariants are defined as follows, respectively:

(16) $$J_{2}^{\prime }=-s_{xx}^{\prime }s_{yy}^{\prime }-s_{yy}^{\prime }s_{zz}^{\prime }-s_{zz}^{\prime }s_{xx}^{\prime }+{s^{\prime }}_{xy}^{2}+{s^{\prime }}_{yz}^{2}+{s^{\prime }}_{xz}^{2}$$

(17) $$J_{3}^{\prime \prime }=s_{xx}^{\prime \prime }s_{yy}^{\prime \prime }s_{zz}^{\prime \prime }+2s_{xy}^{\prime \prime }s_{yz}^{\prime \prime }s_{xz}^{\prime \prime }-s_{xx}^{\prime \prime }{s^{\prime \prime }}_{yz}^{2}-{s^{\prime \prime }}_{yy}{s^{\prime \prime }}_{xz}^{2}-s_{zz}^{\prime \prime }{s^{\prime \prime }}_{xy}^{2}$$

The corresponding fourth-order linear transformation tensor is:

(18) $$L^{\prime }=\left[\begin{array}{cccccc} (c_{2}^{\prime }+c_{3}^{\prime })/3 & -c_{3}^{\prime }/3 & -c_{2}^{\prime }/3 & 0 & 0 & 0\\ -c_{3}^{\prime }/3 & (c_{1}^{\prime }+c_{3}^{\prime })/3 & -c_{1}^{\prime }/3 & 0 & 0 & 0\\ -c_{2}^{\prime }/3 & -c_{1}^{\prime }/3 & (c_{1}^{\prime }+c_{2}^{\prime })/3 & 0 & 0 & 0\\ 0 & 0 & 0 & c_{4}^{\prime } & 0 & 0\\ 0 & 0 & 0 & 0 & c_{5}^{\prime } & 0\\ 0 & 0 & 0 & 0 & 0 & c_{6}^{\prime } \end{array}\right]$$

(19) $$L^{\prime \prime }=\left[\begin{array}{cccccc} (c_{2}^{\prime \prime }+c_{3}^{\prime \prime })/3 & -c_{3}^{\prime \prime }/3 & -c_{2}^{\prime \prime }/3 & 0 & 0 & 0\\ -c_{3}^{\prime \prime }/3 & (c_{1}^{\prime \prime }+c_{3}^{\prime \prime })/3 & -c_{1}^{\prime \prime }/3 & 0 & 0 & 0\\ -c_{2}^{\prime \prime }/3 & -c_{1}^{\prime \prime }/3 & (c_{1}^{\prime \prime }+c_{2}^{\prime \prime })/3 & 0 & 0 & 0\\ 0 & 0 & 0 & c_{4}^{\prime \prime } & 0 & 0\\ 0 & 0 & 0 & 0 & c_{5}^{\prime \prime } & 0\\ 0 & 0 & 0 & 0 & 0 & c_{6}^{\prime \prime } \end{array}\right]$$

where $c_{i}^{\prime }$ and $c_{i}^{\prime \prime }$ (*i* = 1, 2, …, 6) are the anisotropy parameters of the model, which need to be calibrated against experimental data. In the three-dimensional case, the stress can be expressed as:

(20) $$\sigma_{ij}={\left(\begin{array}{cccccc} \sigma_{xx} & \sigma_{yy} & \sigma_{zz} & \sigma_{yz} & \sigma_{xz} & \sigma_{xy} \end{array}\right)}^{T}$$

For the in-plane mechanical behavior of the sheet metal considered in this study, a two-dimensional state is adopted, and the corresponding invariants can be simplified as:

(21) $$J_{2}^{\prime }=\left(\begin{array}{l} \left((c_{2}^{\prime }+c_{3}^{\prime })\sigma_{xx}-c_{3}^{\prime }\sigma_{yy}\right)\left(c_{3}^{\prime }\sigma_{xx}-(c_{1}^{\prime }+c_{3}^{\prime })\sigma_{yy}\right)\\ -\left(c_{3}^{\prime }\sigma_{xx}-(c_{1}^{\prime }+c_{3}^{\prime })\sigma_{yy}\right)\left(c_{2}^{\prime }\sigma_{xx}+c_{1}^{\prime }\sigma_{yy}\right)\\ +\left(c_{2}^{\prime }\sigma_{xx}+c_{1}^{\prime }\sigma_{yy}\right)\left((c_{2}^{\prime }+c_{3}^{\prime })\sigma_{xx}-c_{3}^{\prime }\sigma_{yy}\right) \end{array}\right)/9+{\left(c_{6}^{\prime }\sigma_{xy}\right)}^{2}$$

(22) $$J_{3}^{\prime \prime }=\left((c_{2}^{\prime \prime }+c_{3}^{\prime \prime })\sigma_{xx}-c_{3}^{\prime \prime }\sigma_{yy}\right)\left(c_{3}^{\prime \prime }\sigma_{xx}-(c_{1}^{\prime \prime }+c_{3}^{\prime \prime })\sigma_{yy}\right)\left(c_{2}^{\prime \prime }\sigma_{xx}+c_{1}^{\prime \prime }\sigma_{yy}\right)/27+\left(c_{2}^{\prime \prime }\sigma_{xx}+c_{1}^{\prime \prime }\sigma_{yy}\right){\left(c_{6}^{\prime \prime }\sigma_{xy}\right)}^{2}/3$$

Therefore, the yield function in the 2D case can be rewritten as:

(23) $$f(\sigma )={\left\{\begin{array}{l} {\left[\left(\begin{array}{l} \left(({c^{\prime }}_{2}+c_{3}^{\prime })\sigma_{xx}-c_{3}^{\prime }\sigma_{yy}\right)\left(c_{3}^{\prime }\sigma_{xx}-(c_{1}^{\prime }+c_{3}^{\prime })\sigma_{yy}\right)\\ -\left(c_{3}^{\prime }\sigma_{xx}-(c_{1}^{\prime }+c_{3}^{\prime })\sigma_{yy}\right)\left(c_{2}^{\prime }\sigma_{xx}+c_{1}^{\prime }\sigma_{yy}\right)\\ +\left(c_{2}^{\prime }\sigma_{xx}+c_{1}^{\prime }\sigma_{yy}\right)\left((c_{2}^{\prime }+c_{3}^{\prime })\sigma_{xx}-c_{3}^{\prime }\sigma_{yy}\right) \end{array}\right)/9+{\left(c_{6}^{\prime }\sigma_{xy}\right)}^{2}\right]}^{3/2}\\ -\left[\begin{array}{l} \frac{\left((c_{2}^{\prime \prime }+c_{3}^{\prime \prime })\sigma_{xx}-c_{3}^{\prime \prime }\sigma_{yy}\right)\left(c_{3}^{\prime \prime }\sigma_{xx}-(c_{1}^{\prime \prime }+c_{3}^{\prime \prime })\sigma_{yy}\right)\left(c_{2}^{\prime \prime }\sigma_{xx}+c_{1}^{\prime \prime }\sigma_{yy}\right)}{27}\\ +\left(c_{2}^{\prime \prime }\sigma_{xx}+c_{1}^{\prime \prime }\sigma_{yy}\right){\left(c_{6}^{\prime \prime }\sigma_{xy}\right)}^{2}/3 \end{array}\right] \end{array}\right\}}^{\frac{1}{3}}=\sigma_{eq}$$

To calibrate the model parameters of the above function, the relationship between orientation-dependent stress intensity and stress components in the local and fixed coordinate systems is established. For tension and compression, respectively, we have:

(24) $$\sigma_{xx}=\sigma_{\theta ,T}\cos^{2}\theta ,\sigma_{yy}=\sigma_{\theta ,T}\sin^{2}\theta ,\sigma_{xy}=\sigma_{\theta ,T}\sin\theta \cos\theta$$

(25) $$\sigma_{xx}=-\sigma_{\theta ,C}\cos^{2}\theta ,\sigma_{yy}=-\sigma_{\theta ,C}\sin^{2}\theta ,\sigma_{xy}=-\sigma_{\theta ,C}\sin\theta \cos\theta$$

Substituting the above formulas into Equation (23) yields the orientation-dependent tensile and compressive strengths:

(26) $$\sigma_{\theta ,T}=\frac{\tau_{s}}{{\left[{\left(3{k^{\prime }}_{1}^{2}+{k^{\prime }}_{1}^{2}\right)}^{3/2}+2k_{1}^{\prime \prime }\left({k^{\prime \prime }}_{1}^{2}-{k^{\prime \prime }}_{2}^{2}\right)\right]}^{1/3}}$$

(27) $$\sigma_{\theta ,C}=\frac{\tau_{s}}{{\left[{\left(3{k^{\prime }}_{1}^{2}+{k^{\prime }}_{1}^{2}\right)}^{3/2}-2k_{1}^{\prime \prime }\left({k^{\prime \prime }}_{1}^{2}-{k^{\prime \prime }}_{2}^{2}\right)\right]}^{1/3}}$$

where

(28) $$\begin{array}{l} k_{1}^{\prime }=\frac{c_{2}^{\prime }\cos^{2}\theta +c_{1}^{\prime }\sin^{2}\theta }{6}\\ k_{2}^{\prime }=\sqrt{{\left(\frac{\left(c_{2}^{\prime }+2c_{3}^{\prime }\right)\cos^{2}\theta -\left(c_{1}^{\prime }+2c_{3}^{\prime }\right)\sin^{2}\theta }{6}\right)}^{2}+{\left(c_{6}^{\prime }\sin\theta \cos\theta \right)}^{2}}\\ k_{1}^{\prime \prime }=\frac{c_{2}^{\prime \prime }\cos^{2}\theta +c_{1}^{\prime \prime }\sin^{2}\theta }{6}\\ k_{2}^{\prime \prime }=\sqrt{{\left(\frac{\left(c_{2}^{\prime \prime }+2c_{3}^{\prime \prime }\right)\cos^{2}\theta -\left(c_{1}^{\prime \prime }+2c_{3}^{\prime \prime }\right)\sin^{2}\theta }{6}\right)}^{2}+{\left(c_{6}^{\prime \prime }\sin\theta \cos\theta \right)}^{2}} \end{array}$$

To calibrate the parameters of the above model, data from tension and compression tests at 0°, 45°, and 90°, as well as from equibiaxial tension and compression tests, are employed in this study. The equibiaxial tension/compression data are referenced from the respective formulas $\sigma_{b}=\frac{\sigma_{0}+2\sigma_{45}+\sigma_{90}}{4}$. It should be noted that the data used in this study are referenced from our previously published papers [44,48,49]; therefore, detailed descriptions of the experimental procedures and results are not repeated here. Accordingly, by combining Equations (26)–(28) and incorporating the material orientation, the orientation-dependent tensile and compressive strength expressions can be obtained as follows:

(29) $$\sigma_{0,T}=\frac{3\tau_{s}}{{\left[{\left({c^{\prime }}_{2}^{2}+{c^{\prime }}_{3}^{2}+c_{2}^{\prime }c_{3}^{\prime }\right)}^{3/2}+c_{2}^{\prime \prime }c_{3}^{\prime \prime }(c_{2}^{\prime \prime }+c_{3}^{\prime \prime })\right]}^{\frac{1}{3}}}$$

(30) $$\sigma_{45,T}=\frac{6\tau_{s}}{{\left[{\left({c^{\prime }}_{1}^{2}+c_{1}^{\prime }c_{2}^{\prime }+{c^{\prime }}_{2}^{2}+9{c^{\prime }}_{6}^{2}\right)}^{3/2}+\left(c_{1}^{\prime \prime }c_{2}^{\prime \prime }-6{c^{\prime \prime }}_{6}^{2}\right)(c_{1}^{\prime \prime }+c_{2}^{\prime \prime })\right]}^{\frac{1}{3}}}$$

(31) $$\sigma_{90,T}=\frac{3\tau_{s}}{{\left[{\left({c^{\prime }}_{1}^{2}+c_{1}^{\prime }c_{3}^{\prime }+{c^{\prime }}_{3}^{2}\right)}^{3/2}+c_{1}^{\prime \prime }c_{3}^{\prime \prime }(c_{1}^{\prime \prime }+c_{3}^{\prime \prime })\right]}^{\frac{1}{3}}}$$

(32) $$\sigma_{b,T}=\frac{3\tau_{s}}{{\left[{\left({c^{\prime }}_{1}^{2}+c_{1}^{\prime }c_{2}^{\prime }+{c^{\prime }}_{2}^{2}\right)}^{3/2}+c_{1}^{\prime \prime }{c^{\prime \prime }}_{2}(c_{1}^{\prime \prime }+c_{2}^{\prime \prime })\right]}^{\frac{1}{3}}}$$

(33) $$\sigma_{0,C}=\frac{3\tau_{s}}{{\left[{\left({c^{\prime }}_{2}^{2}+{c^{\prime }}_{3}^{2}+{c^{\prime }}_{2}c_{3}^{\prime }\right)}^{3/2}-c_{2}^{\prime \prime }c_{3}^{\prime \prime }(c_{2}^{\prime \prime }+c_{3}^{\prime \prime })\right]}^{\frac{1}{3}}}$$

(34) $$\sigma_{45,C}=\frac{6\tau_{s}}{{\left[{\left({c^{\prime }}_{1}^{2}+c_{1}^{\prime }c_{2}^{\prime }+{c^{\prime }}_{2}^{2}+9{c^{\prime }}_{6}^{2}\right)}^{3/2}-\left({c^{\prime \prime }}_{1}c_{2}^{\prime \prime }-6{c^{\prime \prime }}_{6}^{2}\right)(c_{1}^{\prime \prime }+c_{2}^{\prime \prime })\right]}^{\frac{1}{3}}}$$

(35) $$\sigma_{90,C}=\frac{3\tau_{s}}{{\left[{\left({c^{\prime }}_{1}^{2}+c_{1}^{\prime }c_{3}^{\prime }+{c^{\prime }}_{3}^{2}\right)}^{3/2}-c_{1}^{\prime \prime }c_{3}^{\prime \prime }(c_{1}^{\prime \prime }+c_{3}^{\prime \prime })\right]}^{\frac{1}{3}}}$$

(36) $$\sigma_{b,C}=\frac{3\tau_{s}}{{\left[{\left({c^{\prime }}_{1}^{2}+c_{1}^{\prime }c_{2}^{\prime }+{c^{\prime }}_{2}^{2}\right)}^{3/2}-c_{1}^{\prime \prime }{c^{\prime \prime }}_{2}(c_{1}^{\prime \prime }+c_{2}^{\prime \prime })\right]}^{\frac{1}{3}}}$$

Based on this, the corresponding objective function (error function) is constructed as:

(37) $$\begin{array}{ll} Error= & (\frac{\sigma_{0,T}^{\exp.}}{\sigma_{0,T}^{\mathrm{pred}.}}-1{)}^{2}+(\frac{\sigma_{45,T}^{\exp.}}{\sigma_{45,T}^{\mathrm{pred}.}}-1{)}^{2}+(\frac{\sigma_{90,T}^{\exp.}}{\sigma_{90,T}^{\mathrm{pred}.}}-1{)}^{2}+(\frac{\sigma_{b,T}^{\exp.}}{\sigma_{b,T}^{\mathrm{pred}.}}-1{)}^{2}\\ & +(\frac{\sigma_{0,C}^{\exp.}}{\sigma_{0,C}^{\mathrm{pred}.}}-1{)}^{2}+(\frac{\sigma_{45,C}^{\exp.}}{\sigma_{45,C}^{\mathrm{pred}.}}-1{)}^{2}+(\frac{\sigma_{90,C}^{\exp.}}{\sigma_{90,C}^{\mathrm{pred}.}}-1{)}^{2}+(\frac{\sigma_{b,C}^{\exp.}}{\sigma_{b,C}^{\mathrm{pred}.}}-1{)}^{2} \end{array}$$

Based on this, the parameter calibration task is transformed into an optimization problem for Equation (37), i.e., when the value of Error is minimized, the resulting optimized parameters correspond to the values to be calibrated. Ideally, the value of Equation (37) would be zero.

### 2.5. Hardening

For the unified constitutive theory describing the full stress state of AA7075-T6, the tensile stress–strain response in the rolling direction (RD) is adopted as the reference equivalent plastic work. The equivalent stress intensities at different levels of plastic work are listed in Table 1. Based on these values and the principle of equivalent plastic work, the strengths of the material under various loading conditions at different equivalent plastic work levels are obtained, as summarized in Table 2. The optimization of Equation (37) is performed using the Levenberg–Marquardt algorithm, and the resulting optimized model parameters are listed in Table 3. For the equivalent stress $\sigma_{eq}$, simplifying the yield function (Equation (23)) to a simple stress state (uniaxial tension) yields:

(38) $$f(\sigma )=\frac{{\left[{\left({c^{\prime }}_{2}^{2}+{c^{\prime }}_{3}^{2}+c_{2}^{\prime }c_{3}^{\prime }\right)}^{3/2}-c_{2}^{\prime \prime }c_{3}^{\prime \prime }(c_{2}^{\prime \prime }+c_{3}^{\prime \prime })\right]}^{\frac{1}{3}}}{3}\sigma_{xx}=\sigma_{eq}$$

**Figure 1:**
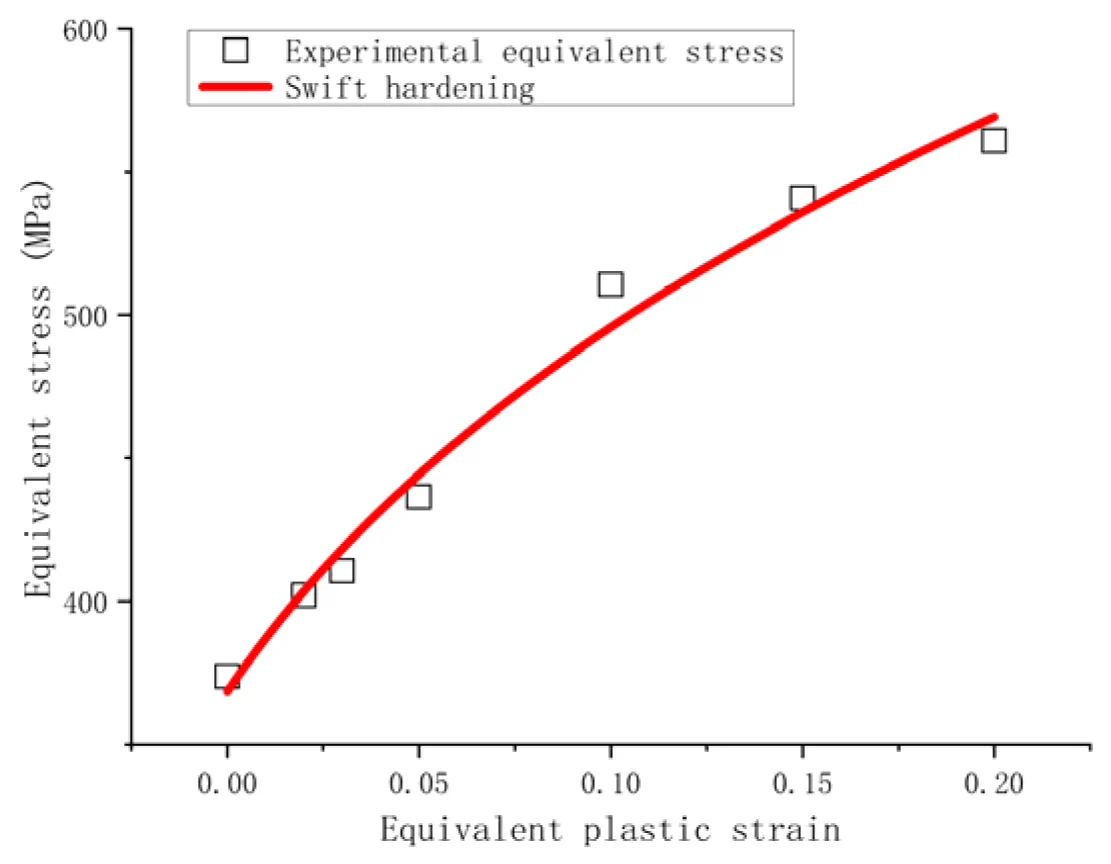
Swift hardening of equivalent stress at different equivalent plastic strains.

The recalculated equivalent stresses are listed in Table 3. To achieve a continuous description of the equivalent stress, the Swift hardening model, as shown in Figure 1, is adopted and described as follows:

(39) $$\sigma_{eq}=828.57(0.0502+\varepsilon_{eq}^{p}{)}^{0.27}$$

The corresponding hardening modulus can then be expressed as:

(40) $${\sigma_{eq}}^{\prime }=223.71{\left(0.0502+\varepsilon_{eq}^{p}\right)}^{-0.73}$$

**Figure 2:**
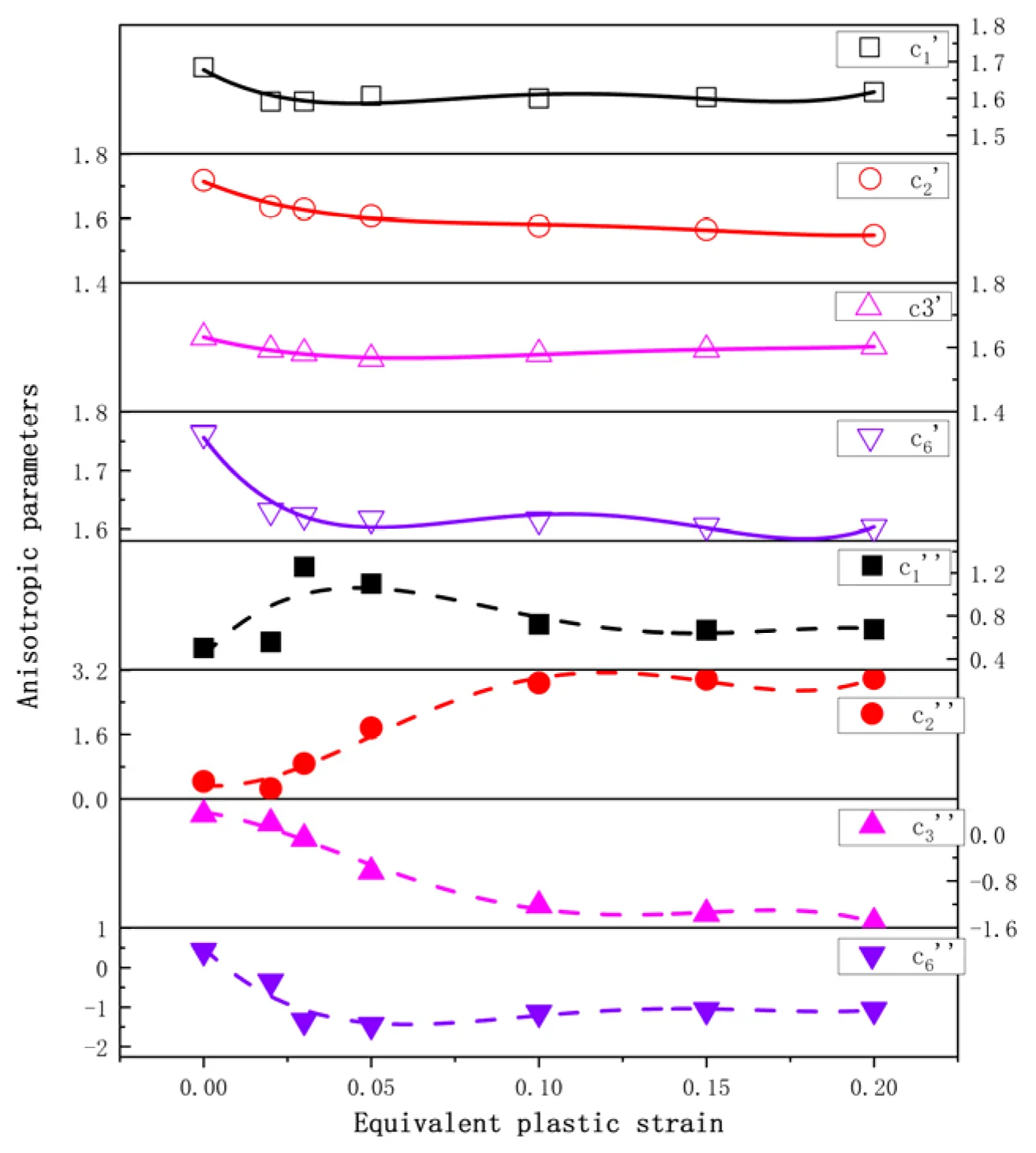
Evolution of anisotropic parameters.

In addition, the Swift hardening of the equivalent stress only captures the trend of yield surface expansion during material deformation and does not account for changes in the shape of the yield surface. The latter is primarily achieved through the evolution of the yield function parameters (Figure 2). The yield criterion can be expressed as:

(41) $$F(\sigma )=f(\sigma )-\sigma_{eq}^{p}$$

By also considering the solution to Equation (13), and given that an associated flow rule is employed, it follows that: $g=f_{\sigma }$ and $g_{\sigma }=f_{\sigma ,\sigma }$. Then:

(42) $$g=f_{\sigma }=\left[\begin{array}{c} f_{1}\\ f_{2}\\ f_{3} \end{array}\right]=\left[\begin{array}{c} f_{\sigma_{xx}}\\ f_{\sigma_{yy}}\\ f_{\sigma_{xy}} \end{array}\right]$$

(43) $$g_{\sigma }=f_{\sigma ,\sigma }=\left[\begin{array}{ccc} f_{\sigma_{xx},\sigma_{xx}} & f_{\sigma_{xx},\sigma_{yy}} & f_{\sigma_{xx},\sigma_{xy}}\\ f_{\sigma_{yy,\sigma_{xx}}} & f_{\sigma_{yy},\sigma_{yy}} & f_{\sigma_{yy},\sigma_{xy}}\\ f_{\sigma_{xy},\sigma_{xx}} & f_{\sigma_{xy},\sigma_{yy}} & f_{\sigma_{xy},\sigma_{xy}} \end{array}\right]$$

Considering the internal variables as shown in Equation (11), the corresponding expression $g_{q}$ can be described as follows:

(44) $$g_{q}=\left[\begin{array}{cccccccc} f_{1,c_{1}^{\prime }} & f_{1,c_{2}^{\prime }} & f_{1,c_{3}^{\prime }} & f_{1,c_{6}^{\prime }} & f_{1,c_{1}^{\prime \prime }} & f_{1,c_{2}^{\prime \prime }} & f_{1,c_{3}^{\prime \prime }} & f_{1,c_{6}^{\prime \prime }}\\ f_{2,c_{1}^{\prime }} & f_{2,c_{2}^{\prime }} & f_{2,c_{3}^{\prime }} & f_{2,c_{6}^{\prime }} & f_{2,c_{1}^{\prime \prime }} & f_{2,c_{2}^{\prime \prime }} & f_{2,c_{3}^{\prime \prime }} & f_{2,c_{6}^{\prime \prime }}\\ f_{3,c_{1}^{\prime }} & f_{3,c_{2}^{\prime }} & f_{3,c_{3}^{\prime }} & f_{3,c_{6}^{\prime }} & f_{3,c_{1}^{\prime \prime }} & f_{3,c_{2}^{\prime \prime }} & f_{3,c_{3}^{\prime \prime }} & f_{3,c_{6}^{\prime \prime }} \end{array}\right]$$

Anisotropic hardening can then be described by the following formula and the vector composed of Equations (16) and (17):

(45) $$h=\left[\begin{array}{cccccccc} f_{c_{1}^{\prime }} & f_{c_{2}^{\prime }} & f_{c_{3}^{\prime }} & f_{c_{6}^{\prime }} & f_{c_{1}^{\prime \prime }} & f_{c_{2}^{\prime \prime }} & f_{c_{3}^{\prime \prime }} & f_{c_{6}^{\prime \prime }} \end{array}\right]$$

In addition, the partial derivative of the flow direction $f_{q}$ with respect to the anisotropic parameters can be described as:

(46) $$f_{q}=\left[\begin{array}{cccccccc} f_{\sigma_{xx,c_{1}^{\prime }}} & f_{\sigma_{xx},c_{2}^{\prime }} & f_{\sigma_{xx},c_{3}^{\prime }} & f_{\sigma_{xx},c_{6}^{\prime }} & f_{\sigma_{xx},c_{1}^{\prime \prime }} & f_{\sigma_{xx},c_{2}^{\prime \prime }} & f_{\sigma_{xx},c_{3}^{\prime \prime }} & f_{\sigma_{xx},c_{6}^{\prime \prime }}\\ f_{\sigma_{yy},c_{1}^{\prime }} & f_{\sigma_{yy},c_{2}^{\prime }} & f_{\sigma_{yy},c_{3}^{\prime }} & f_{\sigma_{yy},c_{6}^{\prime }} & f_{\sigma_{yy},c_{1}^{\prime \prime }} & f_{\sigma_{yy},c_{2}^{\prime \prime }} & f_{\sigma_{yy},c_{3}^{\prime \prime }} & f_{\sigma_{yy},c_{6}^{\prime \prime }}\\ f_{\sigma_{xy},c_{1}^{\prime }} & f_{\sigma_{xy},c_{2}^{\prime }} & f_{\sigma_{xy},c_{3}^{\prime }} & f_{\sigma_{xy},c_{6}^{\prime }} & f_{\sigma_{xy},c_{1}^{\prime \prime }} & f_{\sigma_{xy},c_{2}^{\prime \prime }} & f_{\sigma_{xy},c_{3}^{\prime \prime }} & f_{\sigma_{xy},c_{6}^{\prime \prime }} \end{array}\right]$$

**Figure 3:**
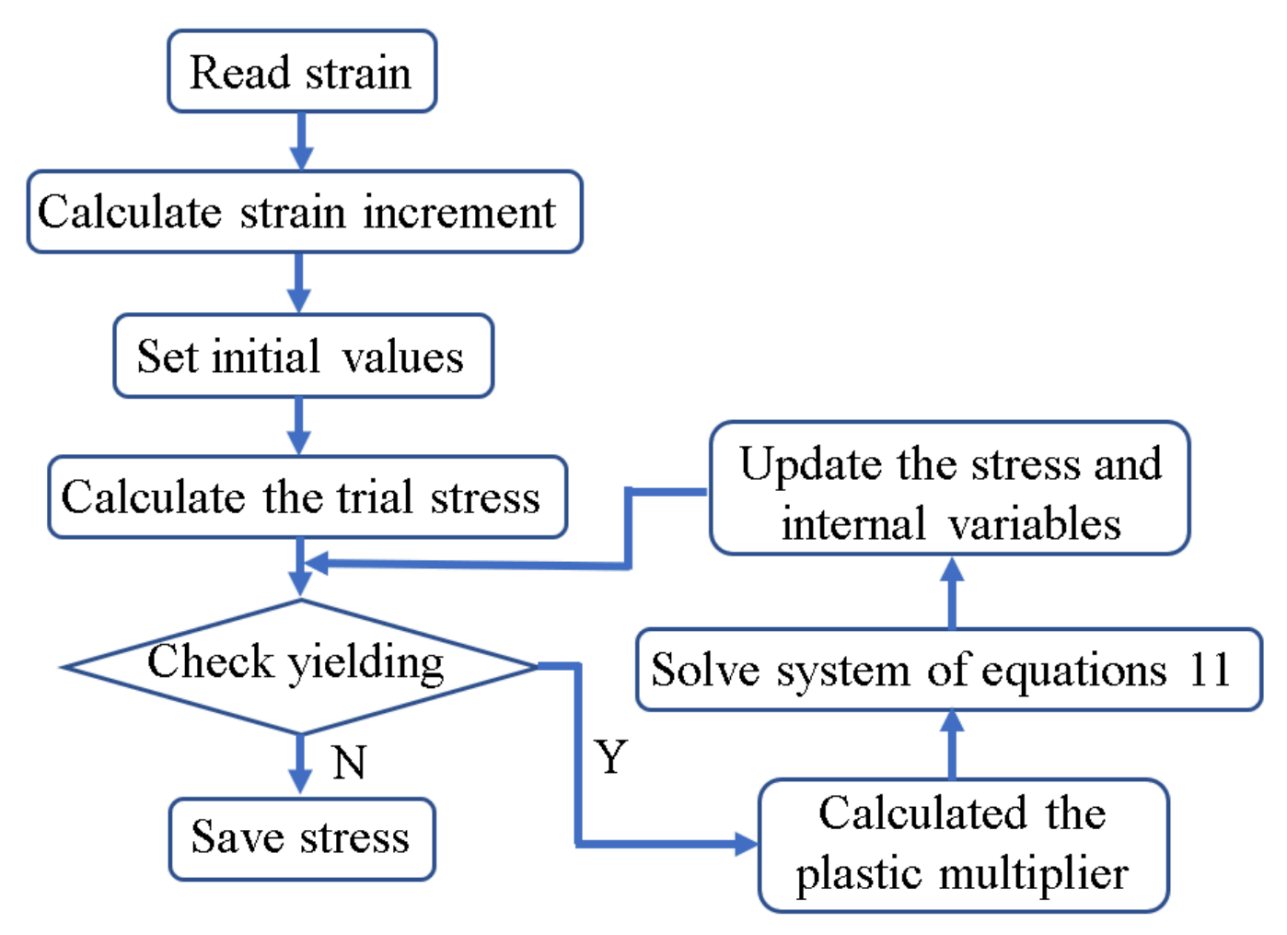
Flowchart of the stress update algorithm.

**Figure 4:**
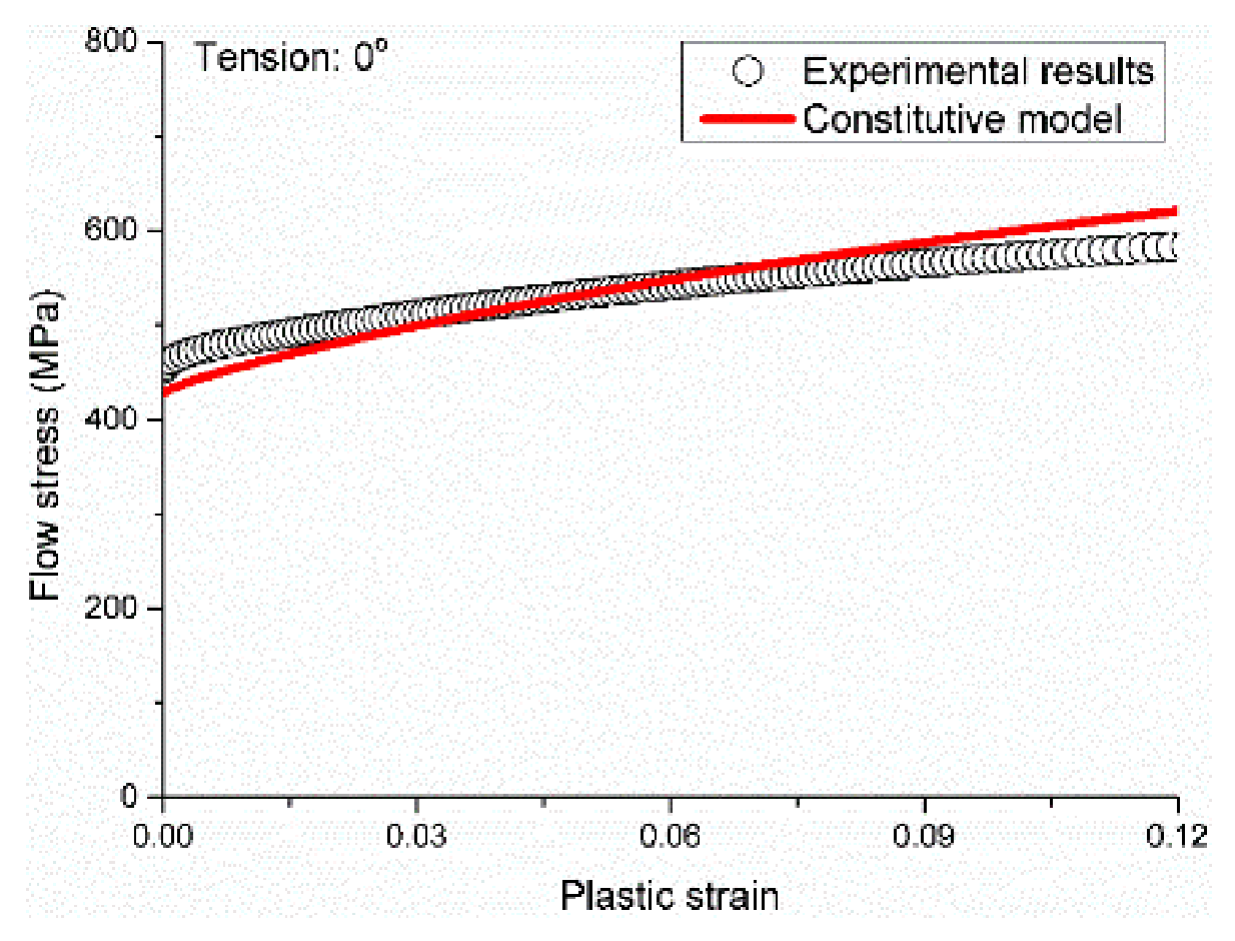
Comparison between stress-strain responses predicted by constitutive model and obtained by experiment.

Based on the above discussion and in conjunction with the stress update algorithm flowchart shown in Figure 3, the stress output at the integration point level under full stress state conditions can be achieved. As illustrated in Figure 4, the stress–strain data predicted by the constructed constitutive model, which accounts for strength asymmetry and incorporates anisotropic hardening behavior, are in good agreement with the corresponding experimental results.

## 3. Machine Learning Model

Problems such as performance prediction and optimization of materials or structures in traditional computational mechanics typically involve solving partial differential equations. Generally, such computational problems are addressed using methods such as meshless methods [50], the finite element method [51], and isogeometric analysis [52]. As demonstrated in the aforementioned work, the anisotropic constitutive model of AA7075-T6, developed based on classical plasticity theory, is often embedded within the finite element framework for problem-solving. For engineering problems, these methods are generally time-consuming and computationally expensive. Furthermore, as noted earlier, although classical plasticity models exhibit good robustness, they suffer from difficulties in modeling, challenges in developing material cards, and limited generalizability. Machine learning models, in contrast, can overcome these issues, provided there is a sufficient dataset with uniformly distributed features for model training. The parallel computing capabilities of computer systems significantly enhance the computational efficiency of machine learning methods while reducing hardware costs, leading to their widespread application in various fields [27,28,29,30,31,33,34,37,53,54,55]. As a specialized type of machine learning, neural networks are primarily inspired by the structure and function of the human brain and are applicable to various types of artificial neural networks (ANNs). For conventional neural networks, the depth is determined by the number of hidden layers between the input and output layers [56]; ANNs with multiple hidden layers are capable of handling highly nonlinear and complex mapping problems. Such characteristics make them well-suited for addressing the unified constitutive behavior of AA7075-T6 under full stress states, enabling the trained model to capture both anisotropic plastic behavior and the strength asymmetry characteristics of the material.

### 3.1. Model Training Strategy

**Figure 5:**
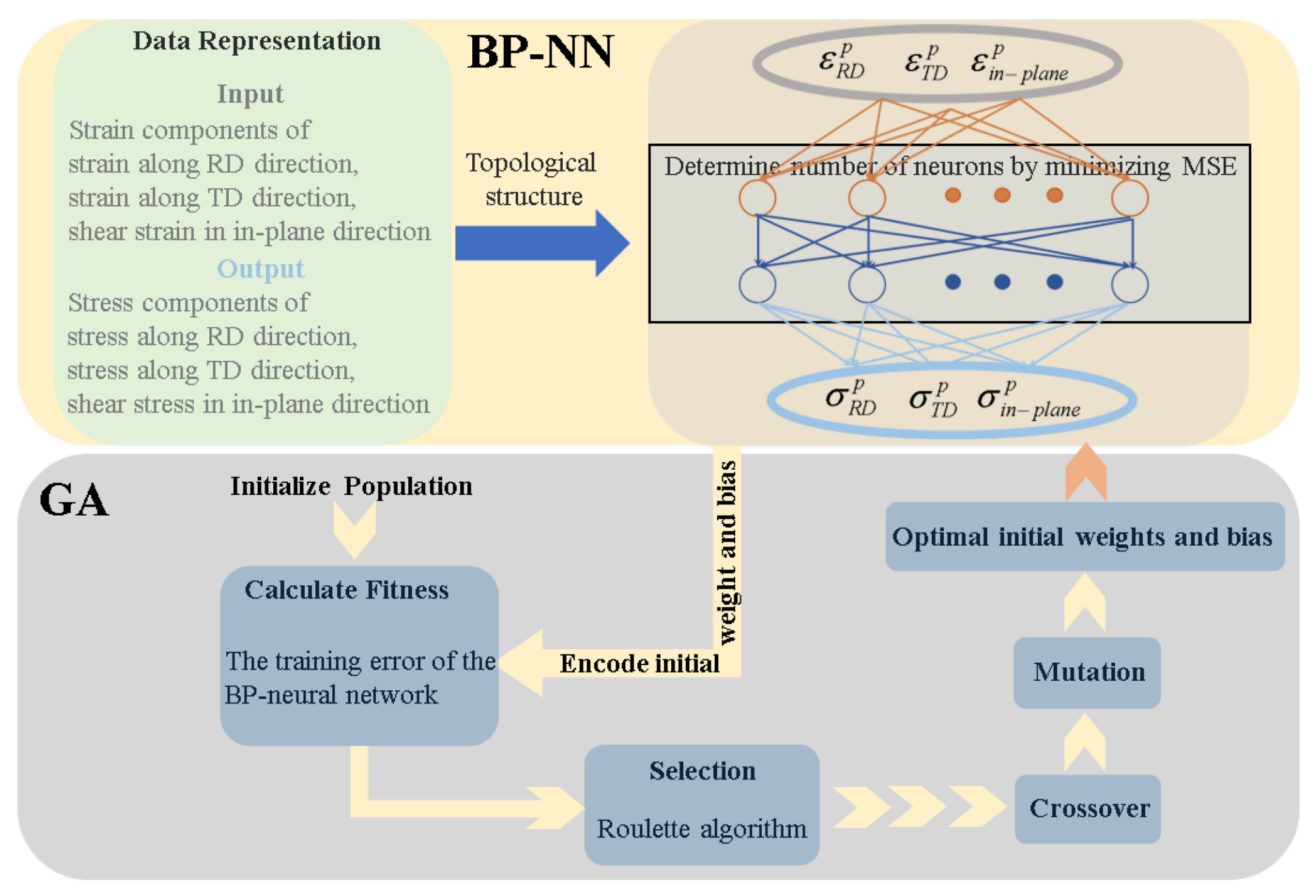
Schematic diagram of machine learning model optimized by GA for mechanical behaviors of AA7075-T6.

To address this, a genetic algorithm-optimized multi-hidden-layer neural network model, as illustrated in Figure 5, is adopted in this study as the machine learning model for predicting the anisotropic mechanical behavior of AA7075-T6. The overall architecture of this model is consistent with that of the previously described machine learning model used for independently predicting the anisotropic tensile, compressive, and shear properties. The main differences lie in the following two aspects: First, the dimensions of the input and output vectors and the reference coordinate systems differ. In the aforementioned machine learning model, the input vector is two-dimensional, considering only the material orientation and the degree of deformation in the local coordinate system, while the output vector considers only the orientation-dependent strength. In contrast, the machine learning model employed here is built based on strain and stress data in the global coordinate system; thus, the input vector consists of three strain components in a unified coordinate system, and the corresponding output vector consists of the three stress components in the same coordinate system. Second, the previously described machine learning model features only one hidden layer, whereas the model in this study utilizes a neural network with two hidden layers to achieve higher nonlinearity and to capture more complex constitutive behavior.

To train the aforementioned machine learning model, this study adopts a strategy that combines experimental results with data predicted by classical plasticity theory to construct the dataset, with the aim of uniformly configuring a dataset with diverse characteristics. For the experimental data from tension, compression, and shear tests at different orientations (0°, 15°, 30°, 45°, 60°, 75°, and 90°), the corresponding strain and stress data are first transformed into the global coordinate system via coordinate transformation. Specifically, the transformation for tension follows Equation (24), for compression follows Equation (25), and for shear follows the following formula:

(47) $$\sigma_{ij}=\left[Q\right]\sigma_{ij}^{\prime }{\left[Q\right]}^{T}=\left|\begin{array}{ccc} 2\sin\theta \cos\theta \tau_{\theta } & (\cos^{2}\theta -\sin^{2}\theta )\tau_{\theta } & 0\\ (\cos^{2}\theta -\sin^{2}\theta )\tau_{\theta } & -2\sin\theta \cos\theta \tau_{\theta } & 0\\ 0 & 0 & 0 \end{array}\right|$$

It should be emphasized here that the same transformation formulas are used for both strain and stress transformations.

The transformed strain and stress data are then numbered accordingly. Specifically, the compression test data at different orientations (0° to 90°) correspond to Data1–Data7 in the dataset; the data generated using the classical plasticity constitutive model (Path 1 to Path 6) correspond to Data8–Data13; the shear test data at different orientations (0° to 90°) correspond to Data14–Data20; and the tensile test data at different orientations (0° to 90°) correspond to Data21–Data27. Each set of data contains the complete loading history for the corresponding loading condition, i.e., the strain and stress components throughout the entire loading process. Moreover, each dataset comprises three independent strain components and three independent stress components, resulting in a dimensionality of six. The first three dimensions correspond to the strain components, which serve as input variables, while the last three dimensions correspond to the stress components, which serve as output variables.

The saved model contains 5171 samples from the 27 curves. The six generated curves were extracted from the archived constitutive histories after removal of initial loading segments and re-referencing of the strain coordinates, and every tenth archived row was retained. Their input columns were exported from the strain variables labelled as plastic strain in the generation scripts. Thus, these training coordinates should not be assumed to be interchangeable with an unprocessed total-strain history. The experimental training curves also include finite flow stresses at zero strain; the resulting mapping is not a general elastic-to-plastic history-dependent constitutive update.

**Figure 6:**
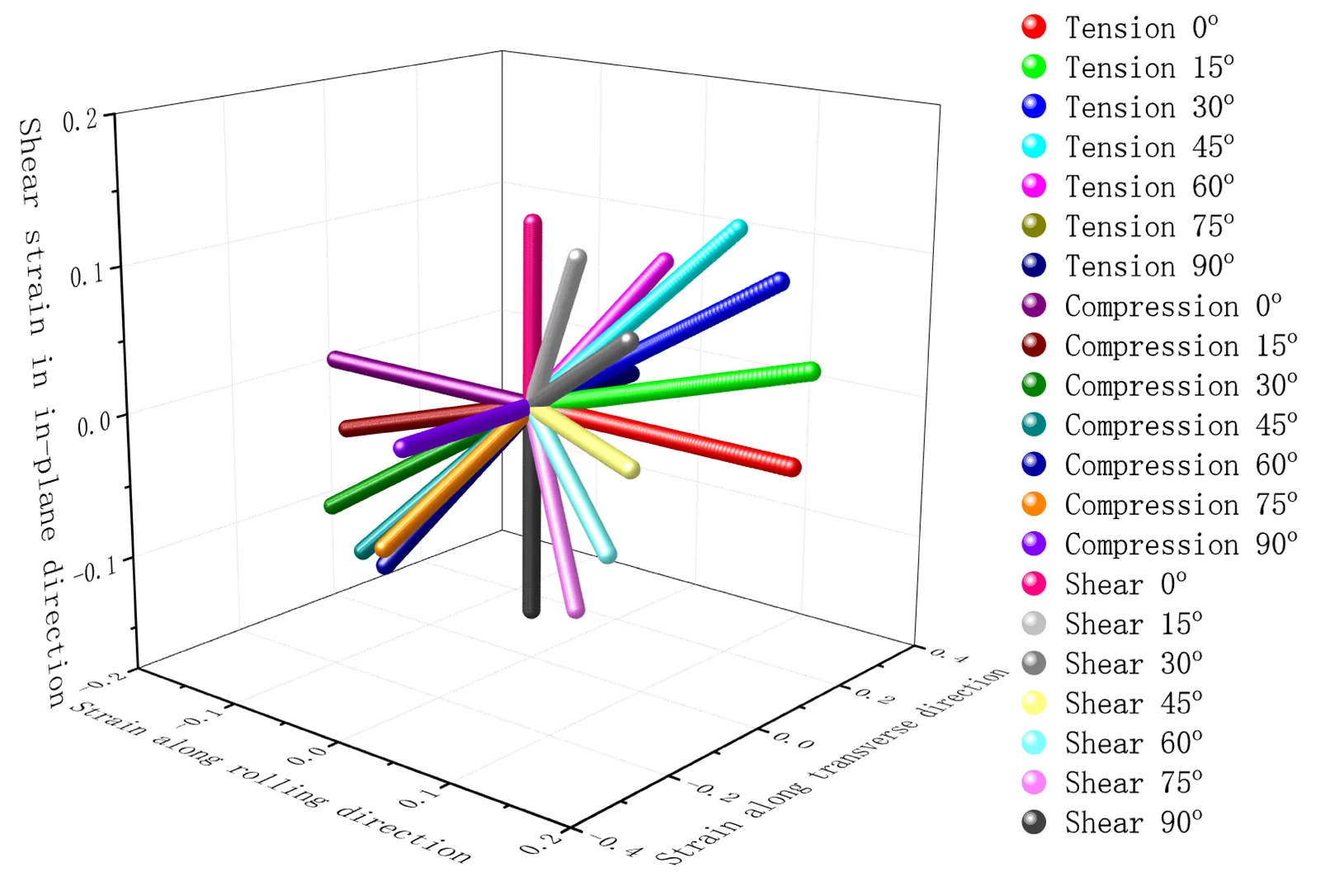
Experimental strain paths (input variables) for training of machine learning model.

**Figure 7:**
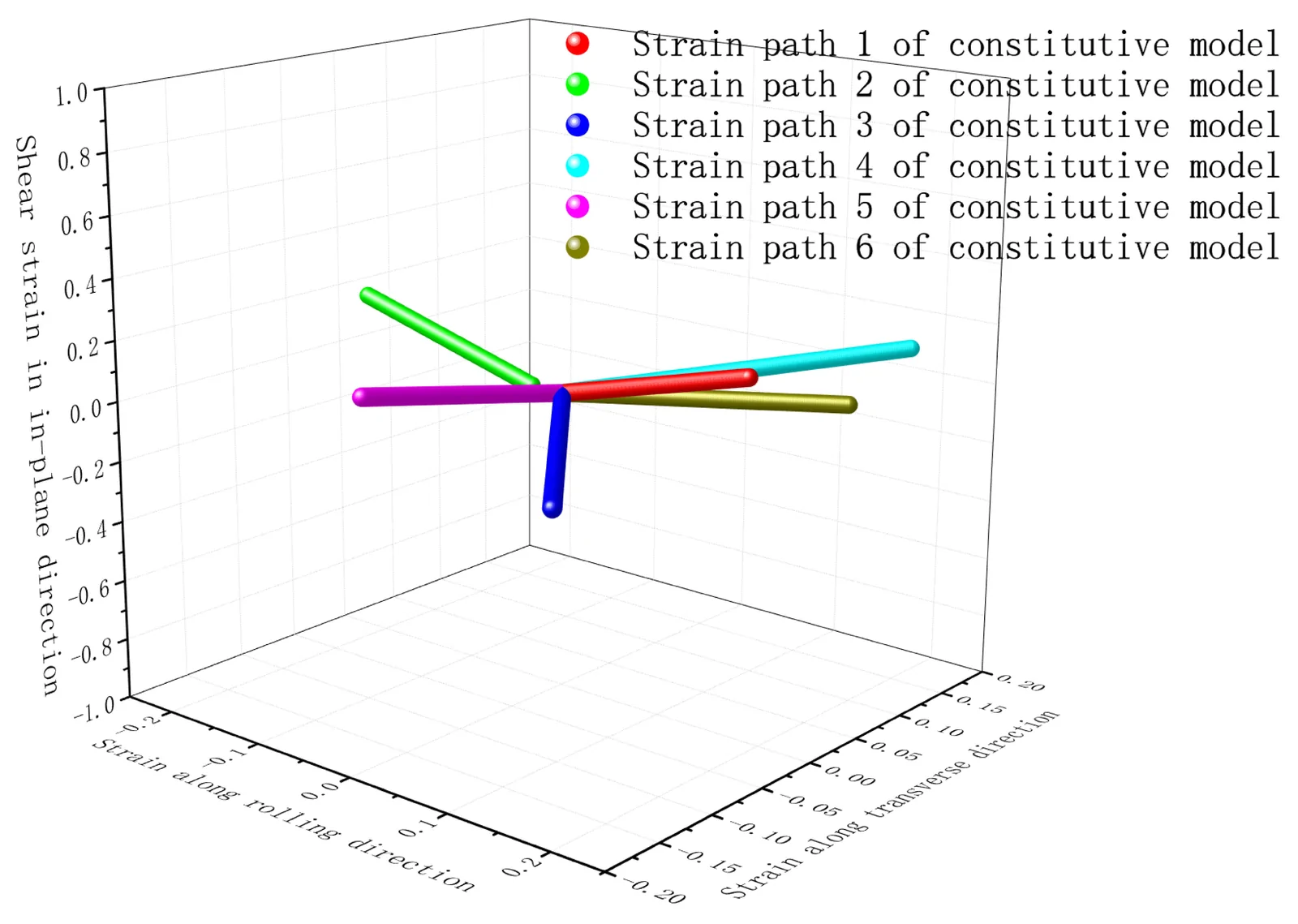
Theoretical strain paths (input variables) for training of machine learning model.

**Figure 8:**
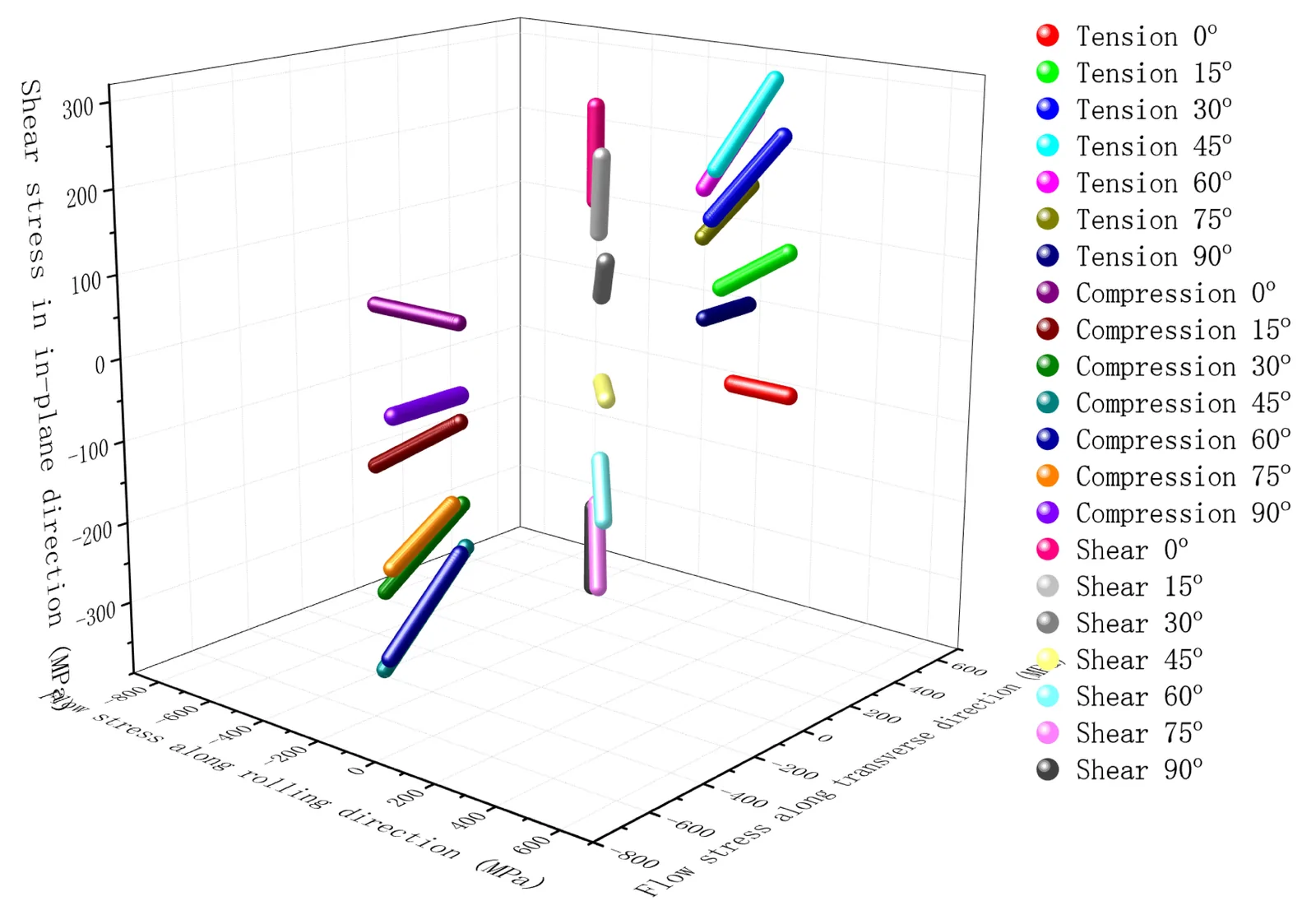
Experimental stress paths (output variables) for training of machine learning model.

**Figure 9:**
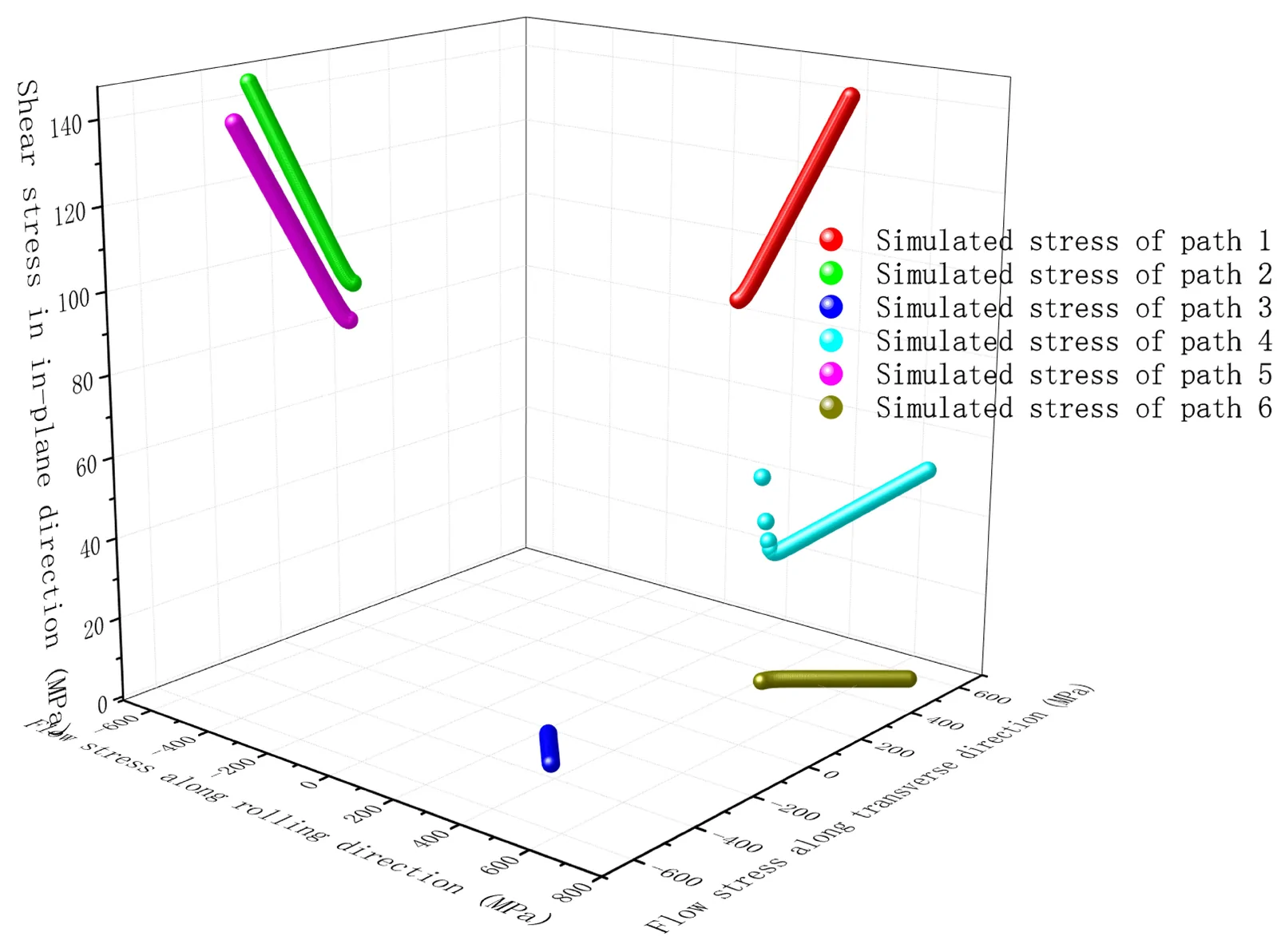
Theoretical stress paths (output variables) for training of machine learning model.

Figure 6 and Figure 7 illustrate the input variables, i.e., the corresponding strain paths, for both the experimental data and the theoretical data used to train the machine learning model, while the corresponding stress paths obtained from experiments and derived from classical plasticity theory are shown in Figure 8 and Figure 9, respectively. It can be observed that the theoretically derived input and output variables effectively complement the characteristics of the experimentally obtained dataset, aiming to further enhance the predictive performance of the trained machine learning model.

**Figure 10:**
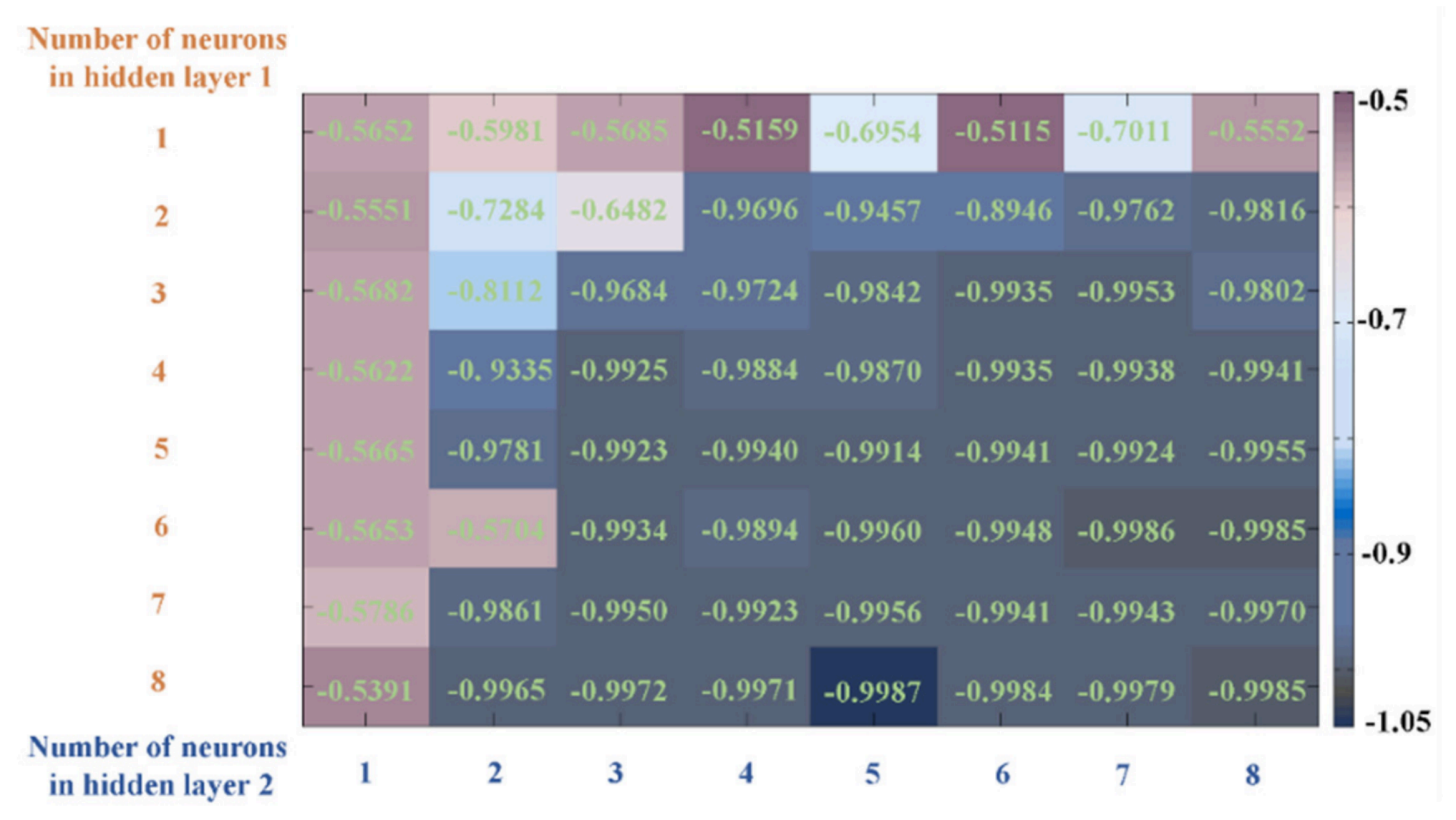
Legacy composite selection score for the 64 combinations of neuron counts in the two hidden layers.

The numbers of neurons in the two hidden layers were selected from 64 combinations, with one to eight neurons in each layer. The quantity shown in Figure 10 is the legacy composite architecture-selection score J = N − C, where N is the MSE of the normalized output and C is the correlation-based scalar returned by the original selection routine. The minimum score was −0.9986625 for the 8–5 architecture. This score can be negative and is neither an MSE nor a conventional coefficient of determination R^2^. The evaluation curve used in this search was also included in the pooled training data; consequently, the search establishes an internal selection preference rather than independent evidence that the 8–5 architecture is optimal for unseen loading histories.

For reproducibility, C is defined by the original routine as

(48) $$\begin{array}{l} C=\frac{\sum_{i}u_{i}d_{i}}{\sum_{i}d_{i}^{2}}\\ \left\{\begin{array}{l} u_{i}=\sum c(T_{ci}-m_{i})/(P_{ci}-v_{i})\\ d_{i}^{2}=\left[{\sum c(T_{ci}-m_{i})}^{2}\right]\left[{\sum c(P_{ci}-v_{i})}^{2}\right]\\ m_{i}=\sum cT_{ci}/(3n)\\ v_{i}=\sum cP_{ci}/(3n) \end{array}\right. \end{array}$$

where T and P denote the reference and predicted stresses, c indexes the three stress components, and n is the number of evaluated points. The centering used in this legacy score differs from conventional Pearson correlation; standard component-wise RMSE and MAE are used for the additional assessment below.

### 3.2. Model Parameters

**Figure 11:**
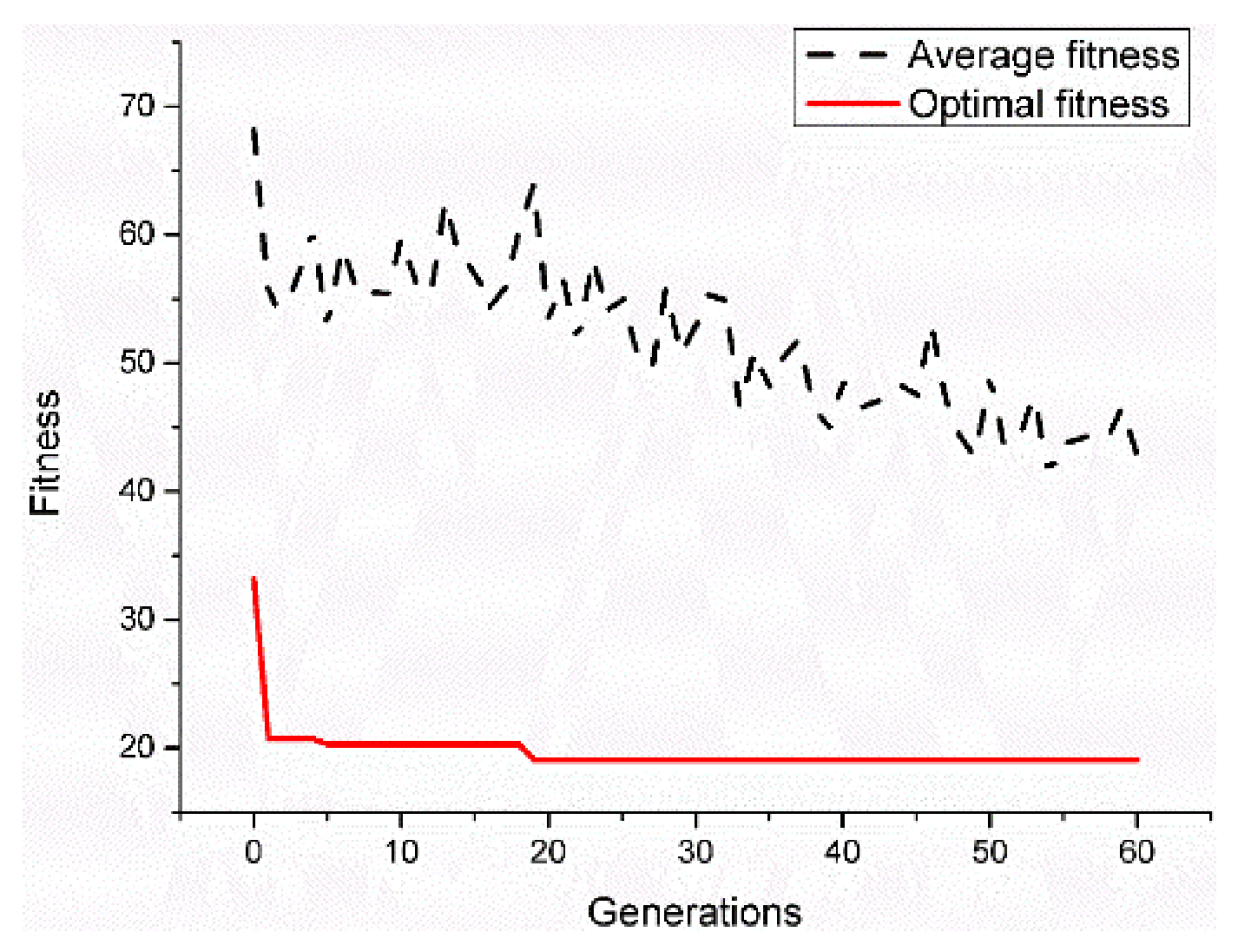
The fitness generations of GA during confirming the parameters of machine learning model.

A genetic algorithm was used to initialize the network weights and biases, followed by backpropagation training. Details of the genetic algorithm are described in our previous work [44,48,49]. Figure 11 shows the evolution of the fitness over 60 generations. Table 4 lists the genetic-algorithm initialization parameters; these are not the final weights after backpropagation. The additional assessment uses the frozen, saved trained network, including its final weights, biases, and input/output normalization parameters.

### 3.3. Model Performance

**Figure 12:**
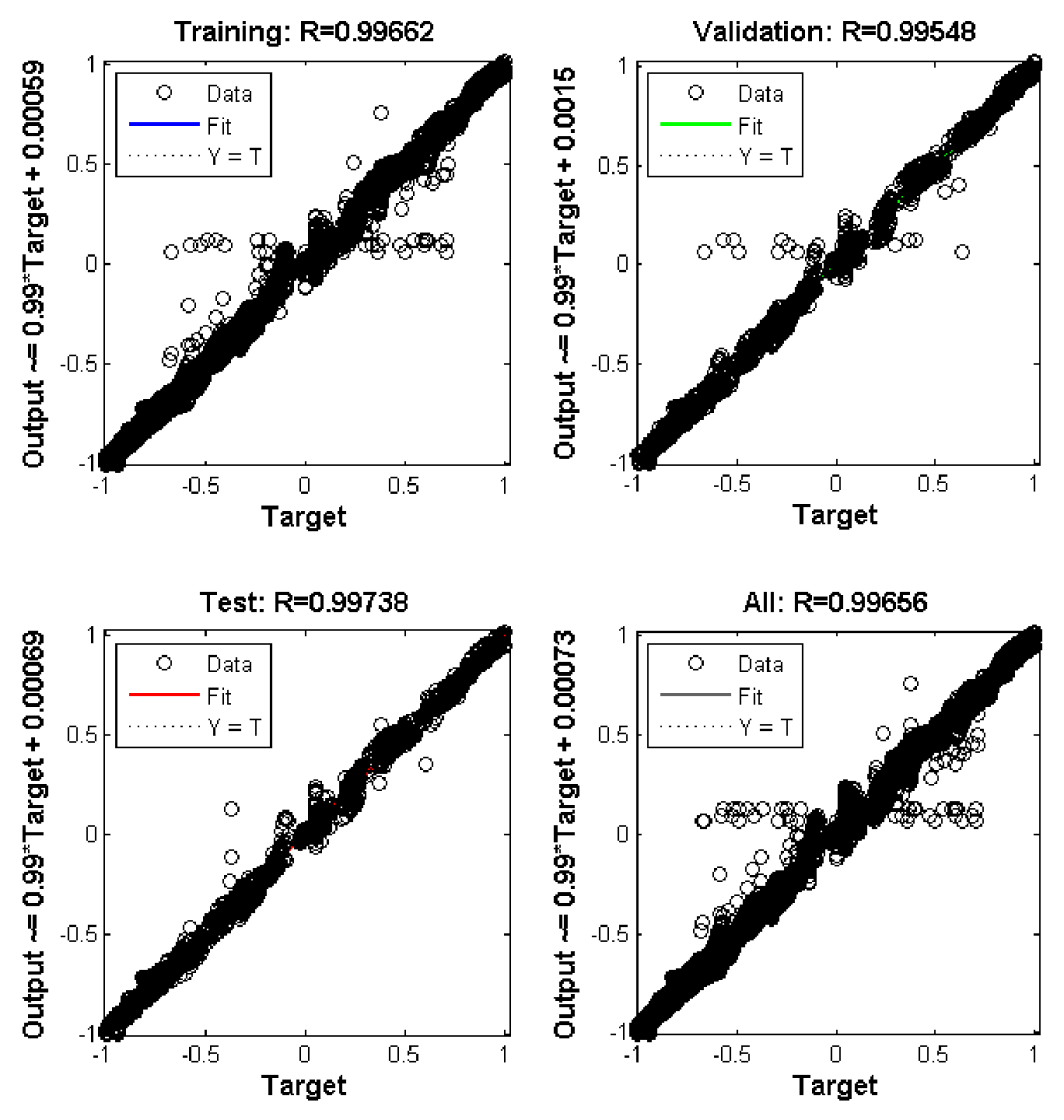
Regression curves of trained machine learning model.

**Figure 13:**
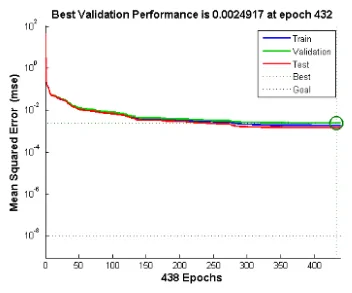
Training performance of machine learning model.

**Figure 14:**
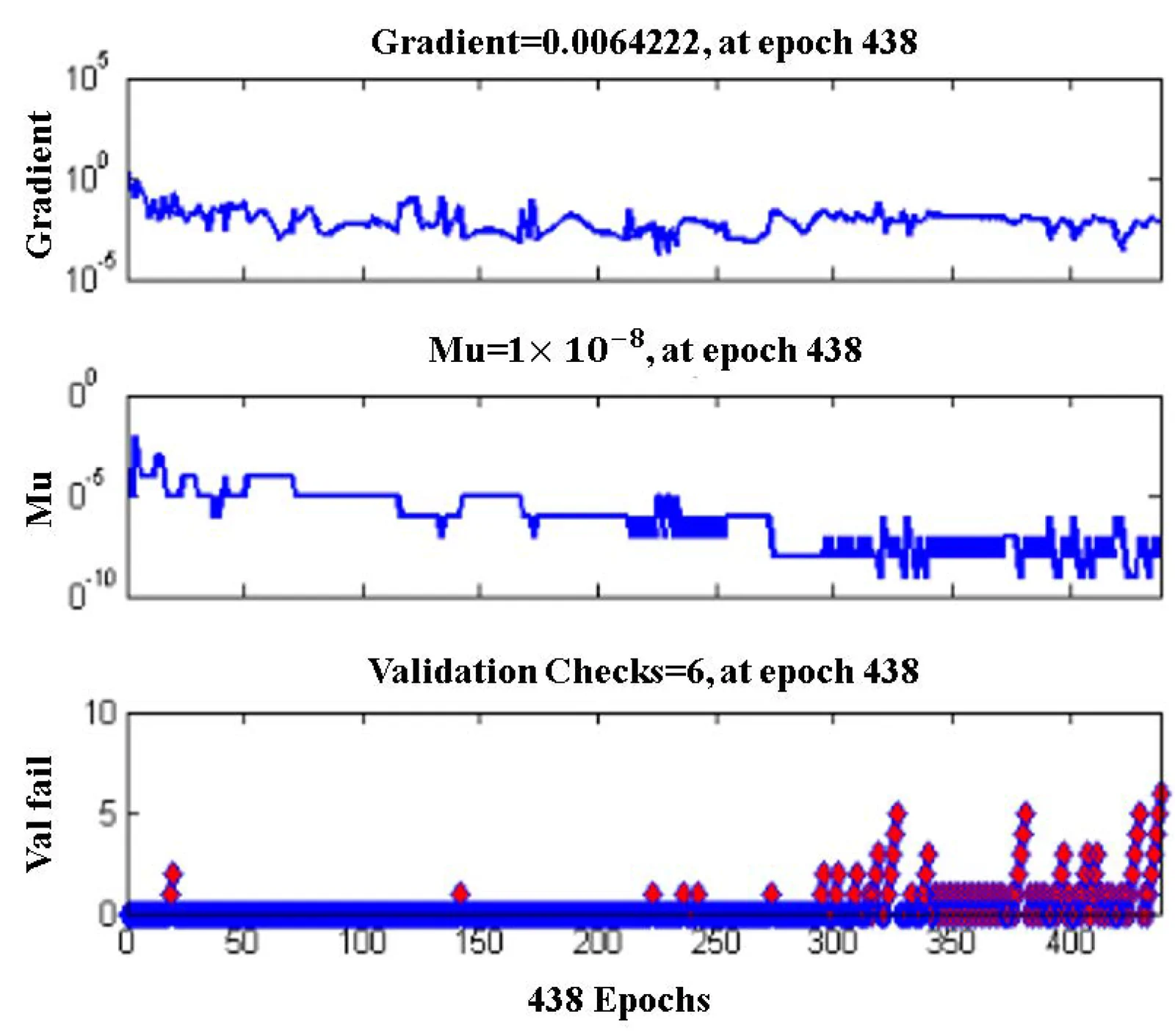
Training states of machine learning model.

The original training, validation, and test regression plots are shown in Figure 12, Figure 13 and Figure 14. Their reported R-values are 0.99662, 0.99548, and 0.99738, respectively, with an overall R-value of 0.99656. The saved model uses random point-wise partitioning in the proportions 70%, 15%, and 15%; complete loading curves were not assigned to mutually exclusive subsets. Normalization was fitted to the pooled dataset before this internal split. These statistics therefore describe internal fit across represented loading families and should not be interpreted as an unbiased test on unseen complete paths. The reported best validation MSE was 0.0025 at epoch 432, and training stopped at epoch 438 after six consecutive validation checks without improvement. Prescribed biaxial plastic strain paths are assessed in Section Assessment of ML Model Under Biaxial Path without retraining or refitting normalization; their relationships to the original training paths are stated explicitly.

## 4. Results and Discussion

**Figure 15:**
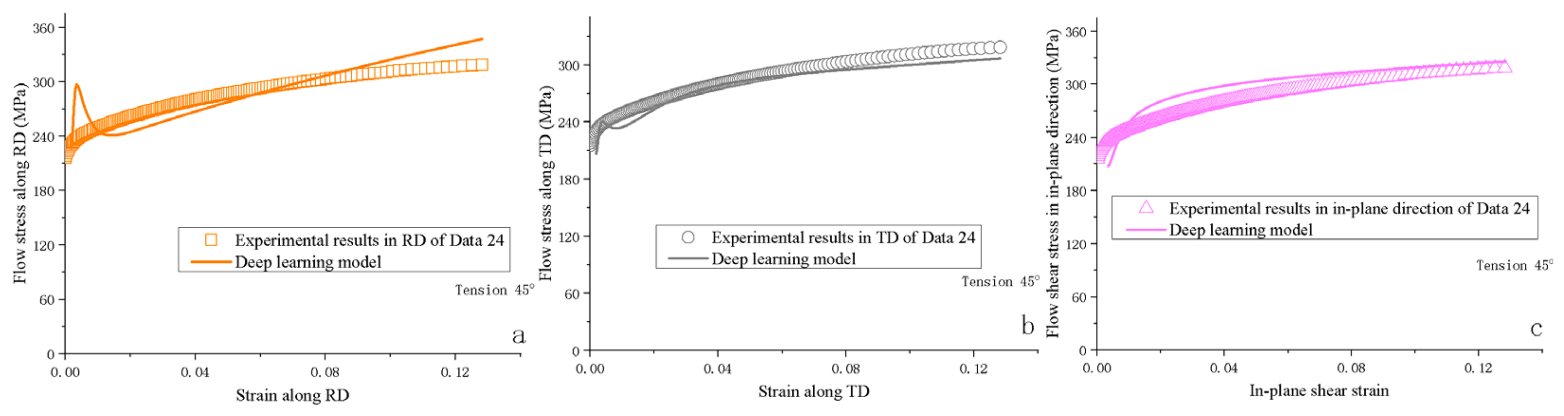
Comparisons between predicted results by machine learning model and experimental data under tension at orientation of 45°, stress along RD (**a**), stress along TD (**b**), and in-plane shear stress (**c**).

**Figure 16:**
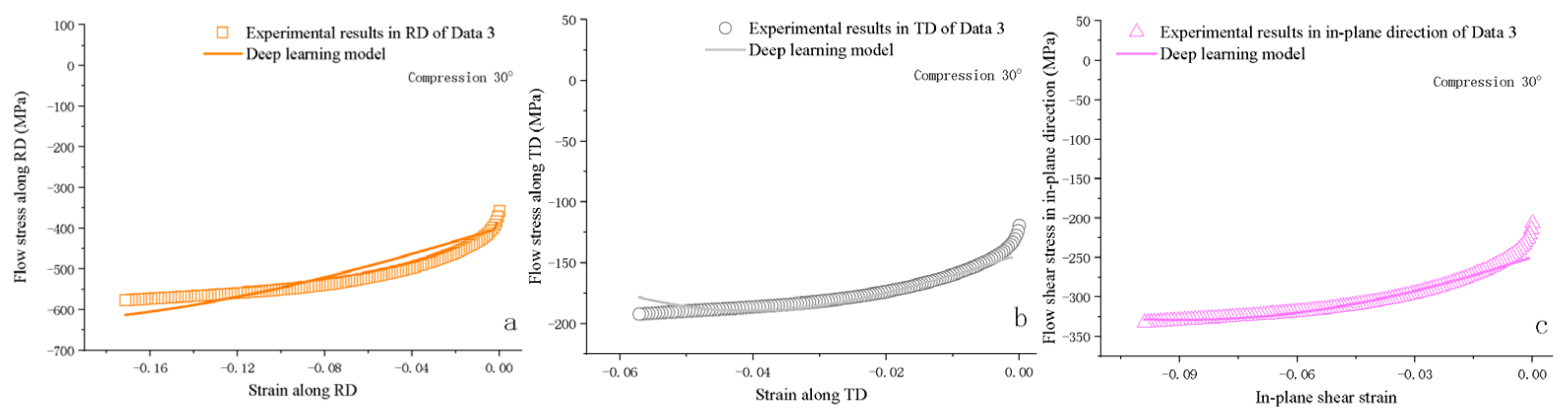
Comparisons between predicted results by machine learning model and experimental data under compression at orientation of 30°, stress along RD (**a**), stress along TD (**b**), and in-plane shear stress (**c**).

**Figure 17:**
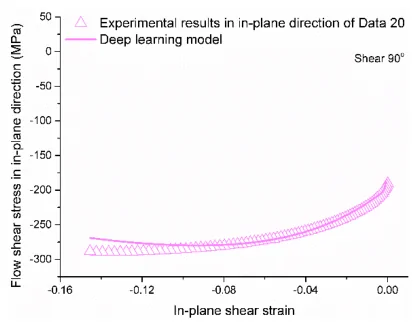
Comparisons between predicted results by machine learning model and experimental data under shear at orientation of 90°.

**Figure 18:**
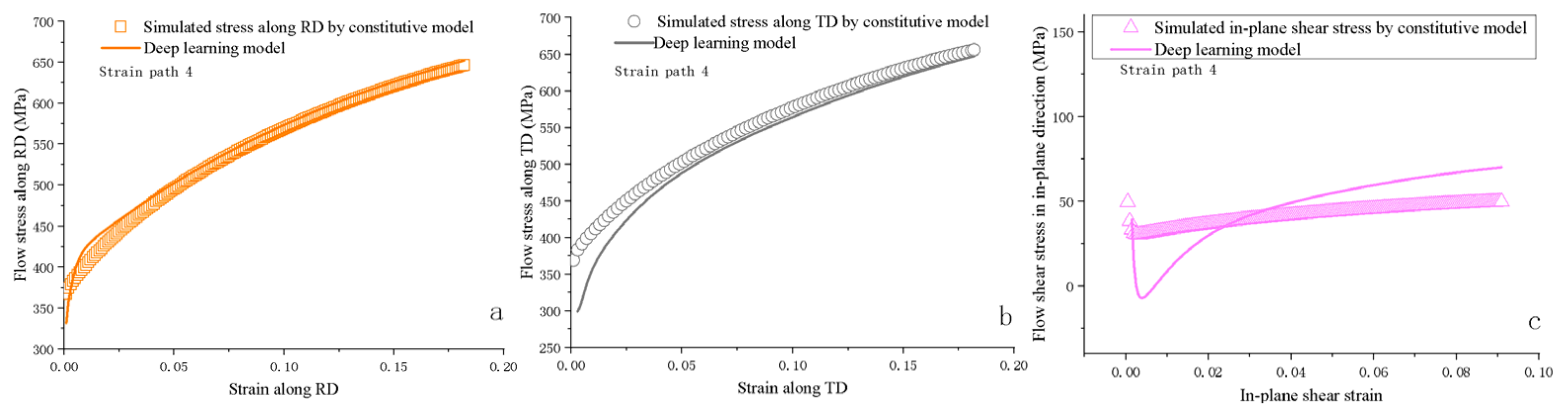
Comparisons between predicted values by machine learning model and theoretical results obtained from constitutive model at strain path 4, stress along RD (**a**), stress along TD (**b**), and in-plane shear stress (**c**).

The comparisons in Figure 15, Figure 16, Figure 17 and Figure 18 assess how the trained network reproduces experimental and constitutive curves represented in the original pooled dataset. They are checks of the fitted strain-to-stress mapping, rather than independent loading-path validation. Section Assessment of ML Model Under Biaxial Path adds normal-stress comparisons under prescribed biaxial plastic strain ratios, distinguishing a represented direction from a direction absent from the archived training inputs.

To address the above requirements, Figure 15, Figure 16 and Figure 17 present comparisons between the predictions of the machine learning model and the corresponding experimental results for tension, compression, and shear, respectively. The material orientation for tension is 45°, while for compression and shear, the orientations are 30° and 90°, respectively. It can be observed from the figures that, in the case of tension, the machine learning model slightly deviates from the experimental results during the initial stage of loading. As loading progresses, the model predictions are in good agreement with the experimental results, particularly for the normal stress in the transverse direction (TD) and the in-plane shear stress. For the normal stress in the rolling direction (RD), the model predictions are slightly higher than the experimental results at the later stage of loading.

For the compression condition at a material orientation of 30° (Figure 16), the stress response predicted by the machine learning model is in good agreement with the experimental results. Only at the initial stage of loading do the predicted values slightly exceed the experimental ones; during the loading process, the predicted stress responses align well with the experimental data. At the later stage of loading, the predicted normal stress in the rolling direction (RD) is slightly higher than the experimental value, whereas the normal stress in the transverse direction (TD) shows the opposite trend, being slightly lower than the experimental value. The predicted in-plane shear stress agrees well with the experimental results.

A comparison between the model predictions and experimental results for the shear condition (90° orientation) is shown in Figure 17. The machine learning model predictions for shear stress at the initial and intermediate stages of loading are nearly identical to the experimental values. Only at the later stage of loading does the model slightly underestimate the shear strength of the material, resulting in a minor deviation from the experimental results.

Figure 18 compares the network output with the constitutive target for strain path 4, which was included in the pooled dataset. The normal stresses are reproduced more closely than the in-plane shear stress. The shear discrepancy is a mismatch with the training reference and cannot be dismissed solely because the shear magnitude is smaller than the normal stresses. This comparison demonstrates reproduction of a represented path but does not establish generalization to an unseen multiaxial strain history or validate a fracture prediction.

### Assessment of ML Model Under Biaxial Path

**Figure 19:**
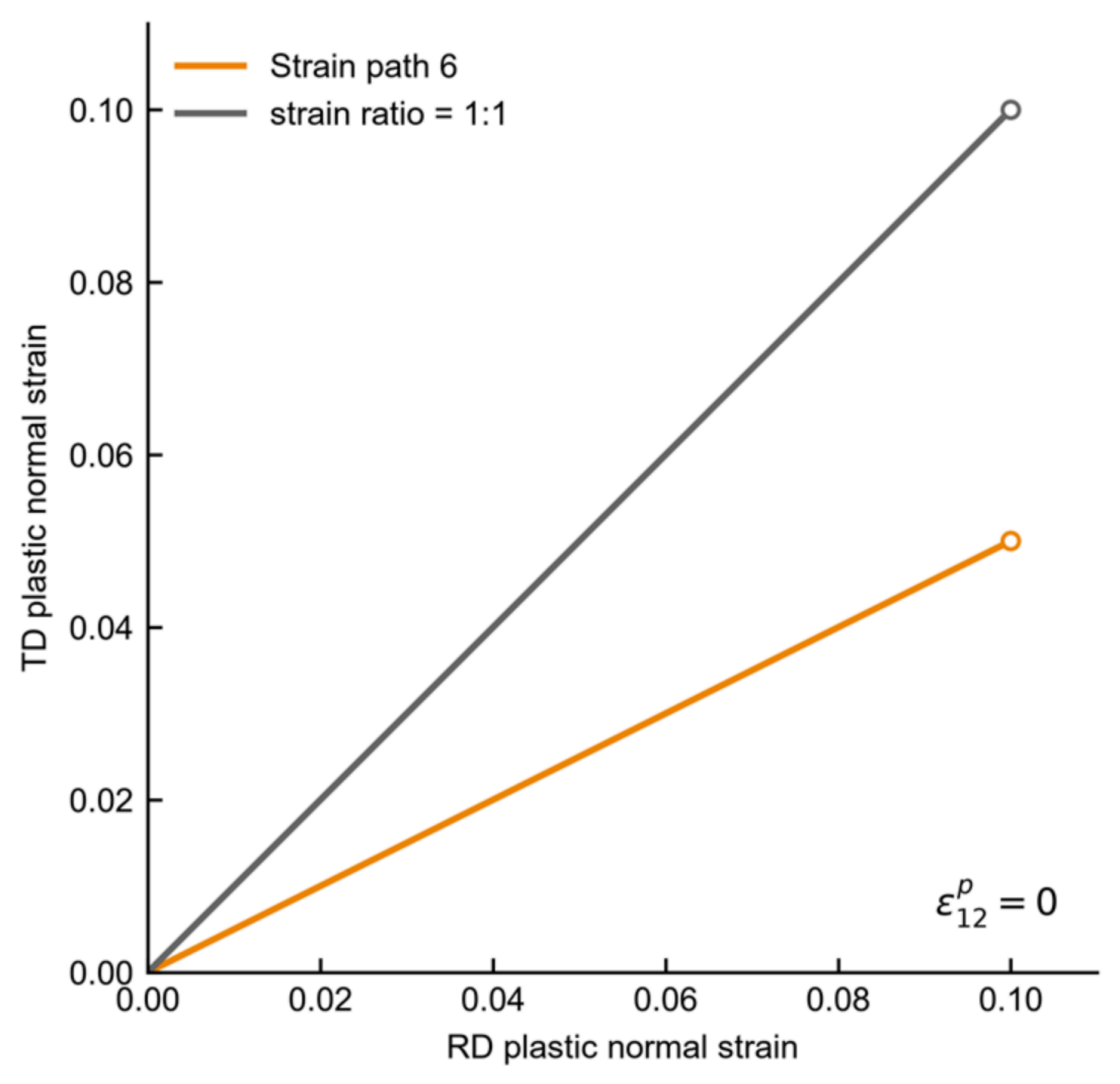
Prescribed plastic strain paths in the fixed RD–TD coordinate system. Strain path 6 follows the sixth constitutive-generated training direction and terminates at RD and TD plastic normal strains of 0.10 and 0.05, respectively. The additional path, labelled strain ratio = 1:1, terminates at (0.10, 0.10). The ratio denotes RD plastic normal strain:TD plastic normal strain. Both paths have zero in-plane plastic shear strain.

Two proportional biaxial tensile calculations were performed with the trained network and its normalization parameters frozen. The first follows Strain path 6, the sixth constitutive-generated loading direction in the training set, with RD and TD plastic normal strains prescribed as p and p/2, respectively. The second uses strain ratio = 1:1, with both plastic normal strains equal to p. Both paths are defined in the fixed RD–TD material coordinate system, with p increasing from zero to 0.10 in increments of 0.00005 and zero in-plane tensor plastic shear strain. Figure 19 shows the inputs. Each comparison includes all 2000 positive-plastic-strain states. The exact zero-input state is excluded because the network represents plastic flow and has a nonzero output at zero input; no correction was subtracted from the remaining network outputs.

The reference stresses were computed using the archived SIM/Swift constitutive implementation. At each increment, the total strain was solved so that the plastic strain, obtained by subtracting the plane-stress elastic strain from the total strain, matched the prescribed input. The elastic compliance used E = 71.7 GPa and a Poisson ratio of 0.33. This operation identifies the plastic strain supplied to both models; no elastic loading branch or additional elastic stress was appended to the network prediction.

**Figure 20:**
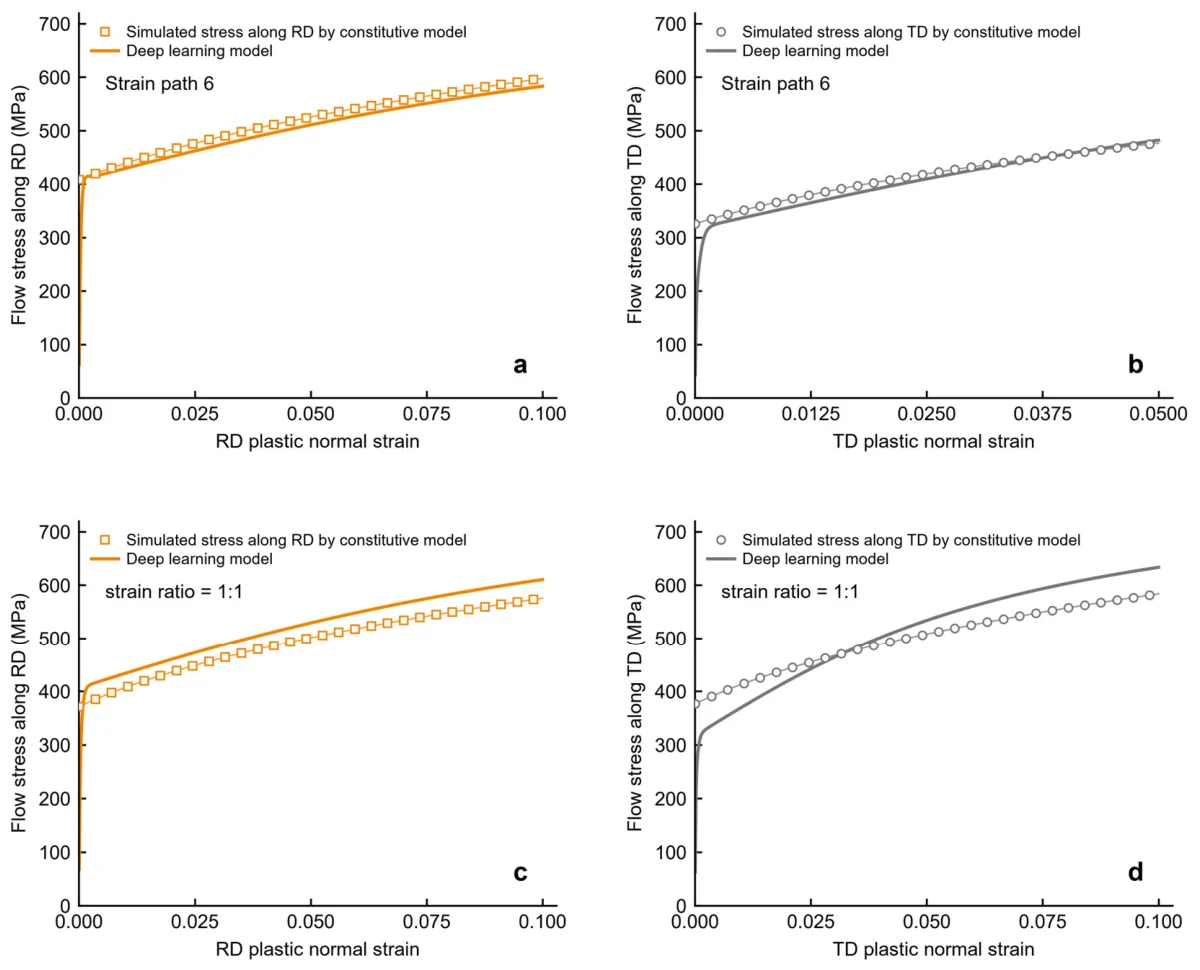
Comparison between normal flow stresses predicted by the deep learning model and calculated using the constitutive model: (**a**) RD and (**b**) TD for Strain path 6; (**c**) RD and (**d**) TD for strain ratio = 1:1.

Figure 20 compares the RD and TD flow stresses against their respective plastic normal strains. For Strain path 6, the normal-stress curves are close over most of the plastic range, although the low-strain mismatch remains visible. The RD and TD RMSE values are 20.35 and 18.67 MPa, respectively, and their mean absolute percentage errors are 2.79% and 2.75%. For strain ratio = 1:1, the corresponding RMSE values increase to 31.87 and 39.24 MPa, with mean absolute percentage errors of 6.11% and 6.90%, as listed in Table 5. At a plastic strain of 0.10, the network overpredicts the RD and TD stresses on the 1:1 path by 6.06% and 8.51%, respectively.

## 5. Conclusions and Remarks

This study combines the respective advantages of classical plasticity theory and machine learning algorithms to establish a machine learning model for the anisotropic mechanical properties of the aerospace lightweight aluminum alloy AA7075-T6. First, a yield model capable of capturing strength asymmetry (i.e., asymmetry between tensile and compressive strengths) and orientation dependence was developed based on a fourth-order linear transformation. Combined with the corresponding hardening law, a complete constitutive model for the material was established. On this basis, a stress update algorithm for the above constitutive model was implemented at the integration point level using the return mapping algorithm and the implicit integration algorithm. In addition to experimental results from tension, compression, and shear tests on specimens with seven different material orientations, additional strain paths covering a more comprehensive stress state were designed. The stress paths corresponding to these designed strain paths were obtained using the developed stress update algorithm. A dataset for the machine learning model of the anisotropic mechanical behavior of AA7075-T6 was constructed by combining these theoretically derived data with the experimental data from tension, compression, and shear tests at various orientations. The dataset was subsequently used to train the machine learning model. The reliability of the trained model was validated by comparing its predictive performance for tension, compression, and shear, respectively. In addition, the results obtained under equi-biaxial tension with a strain ratio of 1:1 assess the predictive capability of the constructed machine learning model for loading directions that were not included in the original training inputs, rather than merely evaluating the material behavior under conditions covered by the training dataset.

## Figures and Tables

**Table 1:** Equivalent stress at different levels of equivalent plastic strain.

$\varepsilon_{eq}^{p}$	0	0.02	0.03	0.05	0.1	0.15	0.2
$W_{eq}^{p}$	0	9.98	14.74	25.28	52.83	81.66	113.27
$\sigma_{eq}^{p}$	453.05	499.57	511.32	532.90	571.73	598.10	619.29

**Table 2:** Stress under various loading conditions at different levels of deformation. All dimensions are in MPa.

$\varepsilon_{eq}^{p}$	$\sigma_{0,C}$	$\sigma_{0,T}$	$\sigma_{45,C}$	$\sigma_{45,T}$	$\sigma_{90,C}$	$\sigma_{90,T}$	$\sigma_{b,C}$	$\sigma_{b,T}$
0	481.63	453.05	469.13	431.28	495.95	450.98	478.96	441.65
0.02	585.50	499.57	573.74	500.31	573.89	518.65	576.72	504.71
0.03	606.40	511.32	591.62	512.00	589.75	530.93	594.85	516.56
0.05	642.06	532.90	621.58	534.49	616.36	553.99	625.40	538.97
0.1	703.55	571.73	675.82	574.34	662.39	592.79	679.39	578.30
0.15	745.17	598.10	711.88	601.53	691.76	617.95	715.17	604.78
0.2	778.23	619.29	739.44	623.18	707.75	638.88	741.21	626.13

**Table 3:** Anisotropic parameters.

$\varepsilon_{eq}^{p}$	$c_{1}^{\prime }$	$c_{2}^{\prime }$	$c_{3}^{\prime }$	$c_{6}^{\prime }$	$c_{1}^{\prime \prime }$	$c_{2}^{\prime \prime }$	$c_{3}^{\prime \prime }$	$c_{6}^{\prime \prime }$	$\sigma_{eq}$/MPa
0	1.68	1.72	1.63	1.76	0.50	0.43	0.33	0.42	373.65
0.02	1.59	1.64	1.59	1.63	0.56	0.26	0.18	−0.36	402.04
0.03	1.59	1.63	1.58	1.62	1.26	0.88	−0.077	−1.35	410.52
0.05	1.61	1.61	1.56	1.62	1.10	1.76	−0.64	−1.45	436.48
0.1	1.60	1.58	1.58	1.62	0.72	2.88	−1.22	−1.14	510.63
0.15	1.60	1.57	1.59	1.61	0.67	2.97	−1.36	−1.07	540.71
0.2	1.62	1.55	1.60	1.60	0.68	2.99	−1.51	−1.07	560.86

**Table 4:** Genetic-algorithm initialization parameters before backpropagation training.

$\omega_{ij}$	−0.23	−2.24	−0.06	1.56	−1.61	−1.87	−4.45	1.69	4.63	−0.77
	4.56	0.12	−1.03	1.79	−0.0047	−0.15	−0.19	1.32	−0.63	0.97
	3.59	0.34	4.83	2.26						
$b_{i}$	0.41	−3.41	−4.32	−0.46	−4.30	−4.36	3.05	−3.36		
$\omega_{ki}$	−0.28	4.46	−3.31	0.17	−2.10	1.04	4.86	−0.91	−2.68	−0.05
	4.39	−2.69	1.44	2.51	−0.46	2.92	−2.10	3.80	3.18	0.39
	−1.34	−3.09	−2.53	0.055	1.76	−0.92	−3.91	−1.62	−2.65	1.77
	−0.47	0.92	2.50	0.62	−1.63	2.17	−4.80	−1.00	1.93	4.73
$b_{k}$	2.54	1.71	−0.98	4.62	−4.15					
$\omega_{lk}$	−0.98	1.39	4.36	−4.73	−4.81	2.68	0.93	4.04	4.35	4.56
	−2.01	−0.51	−1.86	0.97	−0.62					
$b_{l}$	4.93	−2.73	−1.59							

**Table 5:** Normal-stress errors relative to the constitutive implementation for strain ratio = 1:1.

Loading Path	Component	RMSE (MPa)	MAE (MPa)	MAPE (%)
Strain ratio = 1:1	RD	29.82	29.82	6.11
	TD	34.05	34.05	6.90

## Data Availability

The data presented in this study are available on request from the corresponding author due to privacy.
